# Associations of Maternal Use of Benzodiazepines or Benzodiazepine-like Hypnotics During Pregnancy With Immediate Pregnancy Outcomes in Norway

**DOI:** 10.1001/jamanetworkopen.2020.5860

**Published:** 2020-06-22

**Authors:** Anders Huitfeldt, Lene M. Sundbakk, Svetlana Skurtveit, Marte Handal, Hedvig Nordeng

**Affiliations:** 1 Pharmacoepidemiology and Drug Safety Research Group, Department of Pharmacy, PharmaTox Strategic Research Initiative, Faculty of Mathematics and Natural Sciences, University of Oslo, Oslo, Norway; 2 Department of Mental Disorders, Norwegian Institute of Public Health, Oslo, Norway; 3 Department of Child Health and Development, Norwegian Institute of Public Health, Oslo, Norway

## Abstract

**Importance:**

Understanding the safety profile of medications used in pregnancy is crucial for clinical decision-making. Few studies exist on the associations of exposure to benzodiazepines and benzodiazepine-like hypnotic drugs (z-hypnotics) in pregnancy with pregnancy outcomes.

**Objective:**

To determine whether exposure to benzodiazepines or z-hypnotics in pregnancy is associated with greater risk of negative immediate pregnancy outcomes compared with nonexposure.

**Design, Setting, and Participants:**

This questionnaire-based cohort study used data from the Norwegian Mother, Father and Child cohort study (MoBa), which also includes data from the Medical Birth Registry of Norway. Pregnant women were recruited from all over Norway from 1999 and 2008. The first child was born in October 1999 and the last in July 2009. This analysis included women who completed 3 questionnaires, twice during pregnancy and once 6 months after delivery. Data analyses were conducted from September to November 2019.

**Exposures:**

Self-reported exposure to benzodiazepines or z-hypnotics during pregnancy, characterized in terms of any exposure, timing (ie, early, middle, or late), and duration of exposure.

**Main Outcomes and Measures:**

The main outcomes were gestational age at delivery, risk of preterm delivery, birth weight, birth weight relative to gestational age and sex, risk of being small for gestational age, head circumference, Apgar score less than 7 at 5 minutes, and risk of neonatal respiratory distress. Continuous outcomes are reported using effect estimates as mean differences, and binary outcomes are reported using risk ratios.

**Results:**

The MoBa study included 114 234 mother-child dyads. This analysis of MoBa data includes 82 038 singleton pregnancies among 69 434 unique women. Mean (SD) maternal age was 30.2 (4.5) years, and 37 641 pregnancies (45.9%) were in primiparous women. Exposure to benzodiazepines or z-hypnotics was reported in 679 pregnancies (0.8%). After adjusting for all measured baseline and postbaseline confounders, benzodiazepine or z-hypnotic use during pregnancy was associated with lower birth weight (mean difference, −79.3 [95% CI, −126.7 to −31.9] g), lower gestational age at birth (mean difference, −2.1 [95% CI, −3.3 to −0.9] days), and higher risk of preterm birth (risk ratio, 1.41 [95% CI, 1.03 to 1.94]). We found no significant association of exposure to benzodiazepines or z-hypnotics with the child’s birth weight relative to gestational age and sex (*z* score), or any of the other immediate birth outcomes.

**Conclusions and Relevance:**

These findings suggest that the magnitude of the association of exposure to benzodiazepines or z-hypnotics with gestational age is not necessarily clinically significant. The absence of an association of exposure to benzodiazepines or z-hypnotics with *z* score for birth weight relative to gestational age and sex suggests that association of exposure to benzodiazepines or z-hypnotics with birth weight could be explained by earlier delivery rather than impaired intrauterine growth.

## Key Points

**Question:**

Is there an association of prenatal exposure to benzodiazepines or benzodiazepine-like hypnotics with immediate birth outcomes?

**Findings:**

This cohort study including 82 038 pregnancies found that benzodiazepine or benzodiazepine-like hypnotic use during pregnancy was associated with a mean decrease in birth weight of 79 g, a mean decrease in gestational age of 2.1 days, and a 1.41-fold higher risk of preterm birth.

**Meaning:**

While the magnitudes of these findings are not of obvious clinical relevance, benzodiazepines and benzodiazepine-like hypnotic should only be used in pregnancy after a thorough evaluation of the benefits and risks for the mother and child.

## Teaser

This cohort study examines the association of benzodiazepine or benzodiazepine-like hypnotic (z-hypnotics) exposure with immediate birth outcomes among pregnant women in Norway.

## Introduction

Anxiety disorders occur in up to 15% of pregnant women,^1^ and sleep disorders are also prevalent.^2^ Anxiety disorders may require pharmacological treatment, and it has been estimated that 10% to 26% of pregnant women with anxiety disorders are prescribed benzodiazepines or benzodiazepine-like hypnotic drugs (z-hypnotics).^3,4^ In a Norwegian context, up to 1.5% of all pregnant women are prescribed benzodiazepines or z-hypnotics.^5^

When used during pregnancy, these medications cross the placental and blood-brain barrier, where they can bind to γ-amino butyric acid receptors in the developing fetal central nervous system, potentially affecting fetal growth and development.^6,7,8^ An observational study from 2018^9^ and another from 1998^10^ did not detect any significant associations of benzodiazepine exposure with birth weight. However, these medications have been associated with increased risk of preterm birth, low Apgar score, neonatal intensive care unit admission, and respiratory distress syndrome in the infant.^11,12^ Currently, recommendations for use of benzodiazepines in pregnancy are similar to those for women who are not pregnant, meaning that short-term use can be considered for women with severe anxiety disorders or sleep disturbance after an individual risk-benefit evaluation.^13^

We used data from a large population-based cohort study linked with data from a medical birth registry^14^ to examine the association of prenatal exposure to benzodiazepines and z-hypnotics with immediate birth outcomes, including birth weight, gestational age at delivery, *z* score for weight relative to gestational age and sex, head circumference, Apgar score less than 7 at 5 minutes, risk of preterm delivery, and risk of neonatal respiratory distress. While benzodiazepines and z-hypnotics are not first-line treatment for either anxiety or insomnia, determining the outcomes associated with use in pregnancy may be useful for counseling women who enter pregnancy with dependence or who require treatment for clinically significant reasons when other interventions are not working alone.

## Methods

This study was approved by the Regional Committees for Medical and Health Research Ethics, Region South East, Norway. All participants provided written informed consent. Our findings are reported according to the Strengthening the Reporting of Observational Studies in Epidemiology (STROBE) reporting guideline.

### Data Sources and Study Population

This study is based on the Norwegian Mother, Father and Child cohort study (MoBa), a prospective population-based pregnancy cohort study conducted by the Norwegian Institute of Public Health,^15^ and used data from the Medical Birth Registry of Norway (MBRN).^14^ Pregnant women were recruited from all over Norway from 1999 and 2008. The first child was born in October 1999 and the last in July 2009. The women consented to participation in 40.6% of the pregnancies. The cohort now includes 114 500 children, 95 200 mothers, and 75 200 fathers. This study is based on version 9 of the quality-assured data files released for research in 2016. The MBRN is based on mandatory notification of all births or abortions occurring at 12 weeks of gestation or later. It is a nationwide registry that prospectively has collected information on pregnancy, delivery, and the health of the neonate on all births in Norway since 1967.^15^ Follow-up of MoBa participants is conducted by questionnaires at regular intervals and is ongoing. The first MoBa questionnaire 1 (Q1) is completed during week 17 of pregnancy, questionnaire 3 (Q3) is completed during week 30, and questionnaire 4 (Q4) is completed 6 months after birth. Questionnaire 2 is a food frequency questionnaire developed to measure the mother’s diet in pregnancy and completed around week 22 of pregnancy. It was not included in this study.

The establishment of MoBa and initial data collection was based on a license from the Norwegian Data protection agency and approval from the Regional Committee for Medical Research Ethics. The MoBa cohort is currently regulated by the Norwegian Health Registry Act. All mothers and fathers gave informed consent for their questionnaires to be linked with registry data and used for research purposes at the time they enrolled in MoBa.

Of 114 234 pregnancies with records in MoBa and MBRN, 82 038 were included in our primary analyses. Inclusion and exclusion criteria are presented in the Figure. In secondary analyses, we further restricted the population to a disease sample containing 19 554 women who experienced mental health or sleeping issues before or during pregnancy and a benzodiazepines sample of 634 women who used benzodiazepines or z-hypnotics prior to pregnancy.

**Figure.**
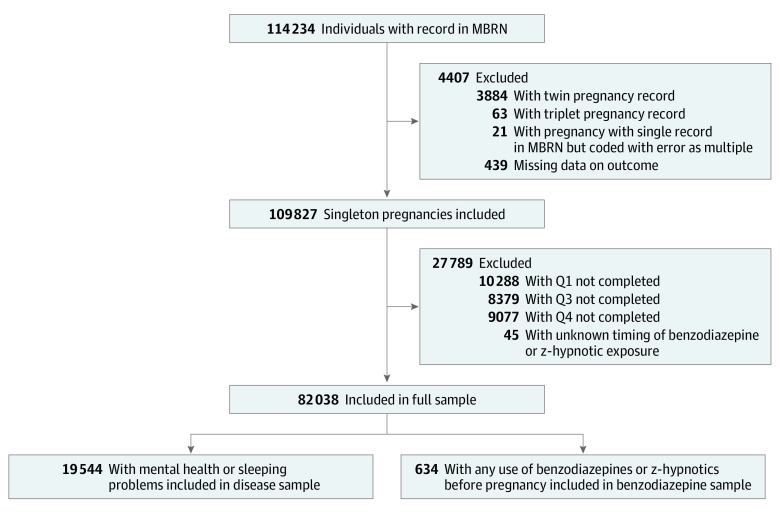
Selection of Study Samples MBRN indicates Medical Birth Registry of Norway; Q, questionnaire; and z-hypnotic, benzodiazepine-like hypnotic drug.

### Exposure

Benzodiazepines included drugs within the Anatomical Therapeutic Chemical Classification System code N05BA (ie, diazepam, oxazepam, and alprazolam), N05CD (ie, nitrazepam, midazolam, and flunitrazepam), and N03AE01 (ie, clonazepam). Z-hypnotics included zopiclone and zolpidem (ie, N05CF). Owing to similar mechanisms of actions, benzodiazepines and z-hypnotics were studied as 1 group and separate classes.

Data on self-reported use of benzodiazepines or z-hypnotics was retrieved from MoBa questionnaires Q1, Q3, and Q4. In the MoBa questionnaires Q1 and Q3, women were asked a range of disease-oriented questions, including whether they have had depression or anxiety or other mental disorders before and/or during pregnancy or sleeping problems during early pregnancy (Q1 only). For each condition, mothers provide information on whether medications were used in 4-week intervals during pregnancy (eg, week 0-4, week 5-8). In the analysis that assessed overall associations of the medications, we classified exposure in terms of whether use of any of these medications was reported in any interval during pregnancy. To examine the relevance of timing, we further categorized exposure according to whether medication use was reported in early pregnancy (ie, weeks 0-16), midpregnancy (ie, weeks 17-28), or late pregnancy (ie, week 29 to delivery). These categories correspond roughly to the MoBa questionnaires on which the relevant items were included but with some overlap, as items for weeks 13 to 16 were included on Q1 and Q3. To explore possible cumulative dose-response association, we classified exposure according to the number of 4-week intervals during which use was reported (ie, no exposure, exposure in 1 interval, exposure in 2 intervals, and exposure in ≥3 intervals). In secondary analyses, we restricted the exposure definition to benzodiazepines (ie, N05BA, N05CD, and N03AE01), to benzodiazepine-anxiolytics only (ie, N05BA), or to z-hypnotics only (ie, N05CF).

### Outcomes

Outcome data was retrieved from MBRN, including gestational age (days), birth weight (grams), birth weight relative to gestational age and sex (*z* score), head circumference (centimeters), Apgar score at 5 minutes, and respiratory distress. These variables are recorded in electronic medical records by midwives at birth and sent to the MBRN. Gestational age at delivery was estimated based on second trimester ultrasonographic results. Information on last menstrual period was used if an ultrasonographic investigation had not been conducted. For analyses of binary outcomes, preterm birth was defined as delivery at less than 37 completed weeks of pregnancy, low Apgar score was defined as score less than 7 at 5 minutes, and small for gestational age was defined as *z* score less than −1.28, which corresponds to the tenth percentile in weight relative to gestational age and sex. All outcomes were prespecified.

### Covariates

Confounders were selected in accordance with the modified disjunctive cause criterion.^16^ We selected the same confounders for all exposure-outcome associations. Baseline covariates selected from MBRN included maternal age at delivery, parity, marital status, maternal education, sex of the child, and folic acid supplements. Baseline covariates selected from MoBa included body mass index before conception, smoking status, illicit drug use, alcohol intake, planned pregnancy, income, ongoing or completed education, adverse life events, sleeping and mental health issues, anxiety and lifetime history of major depression. The mother’s lifetime history of major depression was reported according to 5 key depressive symptoms, which correspond closely to the *Diagnostic and Statistical Manual of Mental Disorders* (Third Edition) criteria for lifetime major depression.^17,18^

Time-varying covariates, all from MoBa, included maternal symptoms of depression and anxiety during pregnancy, comedication use during pregnancy (ie, nonsteroidal anti-inflammatory drugs, opioids, paracetamol, antidepressants, antipsychotics, and antiepileptics), and fever during pregnancy. Maternal symptoms of depression and anxiety during pregnancy were assessed with a validated short version of the Hopkins Symptom Checklist^19^ at gestational week 17 and at week 30, and standardized *z* scores were computed at each time point.

### Missing Data

We accounted for missing data using a combination of multiple imputation and inverse probability of censoring weights.^20,21^ The full procedure for handling missing data relied on an assumption that the data were missing at random and is described in eAppendix 1 in the Supplement.

### Statistical Analysis

We used R statistical software version 3.4.4 (R Project for Statistical Computing) for all statistical analysis. *P* values were 2-sided, and statistical significance was set at .05. We described the distribution of baseline characteristics and the absolute risks and distributions of outcomes in women exposed and unexposed to benzodiazepines or z-hypnotics in pregnancy. For every outcome, we conducted all analyses separately for all 3 definitions of the exposure (ie, ever vs never, timing of exposure, and duration of exposure). We conducted a crude analysis to obtain unadjusted estimates with 95% CIs. In the first adjusted analysis, we controlled only for baseline covariates using standard multivariable regression models. In the second adjusted analysis, we additionally accounted for time-varying covariates using stabilized inverse probability of treatment weights.^22,23^ Linear outcome models were used for continuous outcomes, and log-binomial models were used for binary outcomes. No interaction terms were included in the models. In adjusted analyses, we used sandwich variance estimators that are robust to clustering.^24^ Estimation of the inverse probability of treatment weights and the details of the outcome model are described in eAppendix 2 in the Supplement. No adjustments were made for multiple comparisons.

We also conducted all analyses in the disease sample. In the benzodiazepine sample, we conducted a simplified analysis comparing only women who reported any use of benzodiazepines or z-hypnotics in pregnancy vs women who reported no use in pregnancy. To check for effect modification, we conducted analyses stratified on the sex of the offspring. To explore the robustness of our findings to between-unit clustering, we further conducted analyses in a data set that was restricted to women who participated in MoBa for the first time. We also conducted analyses in which the exposure definition was limited to benzodiazepines, to benzodiazepine anxiolytics only, and to z-hypnotics. Data analyses were conducted from September to November 2019.

## Results

### Characteristics of the Study Population

The study population included 82 038 singleton pregnancies in women who completed all 3 MoBa study questionnaires. The mean (SD) maternal age was 30.2 (4.5) years, 37 641 women (45.9%) were primiparous, and 41 987 infants (51.2%) were boys. Missing data for 82 038 women who completed all questionnaires included prepregnancy body mass index (1992 women [2.4%]), maternal education (345 women [0.4%), smoking (1066 women [1.3%]), alcohol intake (6739 women [8.2%]), income (2614 women [3.2%]), planned pregnancy (119 women [0.1%]), lifetime history of major depression (2012 women [2.5%]), and depressive or anxiety symptoms during pregnancy (2621 women [3.1%] on Q1; 6220 women [7.6%] on Q3).

Among 82 038 women, 679 were exposed to benzodiazepines or z-hypnotics during pregnancy. Women who were exposed to benzodiazepines or z-hypnotics compared with women who were not exposed were older (mean [SD] age, 31.2 [4.8] years vs 30.2 [4.5] years), less likely to be married or cohabiting (614 women [90.4%] vs 78 483 women [96.5%]), more likely to be primiparous (356 women [52.4%] vs 37 285 women [45.8%]), more likely to smoke (112 women [16.5%] vs 6061 women [7.4%]), more likely to report low or moderate alcohol intake during pregnancy (28 women [4.1%] vs 2050 women [2.5%]), more likely to report illicit drug use (33 women [4.9%] vs 468 women [0.6%]), less likely to have a planned pregnancy (189 women [27.8%] vs 66 691 women [82.0%]), more likely to have depression (211 women [31.1%] vs 5328 women [6.5%]), and more likely to have had at least 1 painful or very painful adverse life event (387 women [57.0%] vs 28 377 women [34.9%]). An overview of the distribution of baseline covariates in the exposed and in the unexposed groups is shown as Table 1. Unadjusted data on absolute risks and outcome distributions in the exposed and in the unexposed are shown as Table 2.

**Table 1.** Maternal Characteristics Stratified by Use of Benzodiazepines or Z-Hypnotics During Pregnancy

Variable	Benzodiazepine or z-hypnotic exposure during pregnancy, No. (%)	
	Yes (n = 679)	No (n = 81 359)
Age, mean (SD), y	31.2 (4.8)	30.2 (4.5)
Married or cohabiting with father	614 (90.4)	78 483 (96.5)
Primiparous	356 (52.4)	37 285 (45.8)
Prepregnancy BMI, mean (SD)	23.7 (4.2)	24 (4.2)
Missing	13 (1.9)	1979 (2.4)
College or university education^a^	239 (35.2)	27 847 (34.2)
Missing	<5	343 (0.4)
Smoking	112 (16.5)	6061 (7.4)
Missing	<5	1062 (1.3)
Alcohol intake during pregnancy^b^		
None or minimal	510 (75.1)	72 565 (89.2)
Low to moderate	28 (4.1)	2050 (2.5)
Frequent	<5	70 (0.1)
Missing	64 (9.4)	6675 (8.2)
Gross yearly income, $		
≤17 500	71 (10.5)	7785 (9.6)
17 501-46 800	510 (75.1)	62 157 (76.4)
>46 800	76 (11.2)	8825 (10.8)
Missing	22 (3.2)	2592 (3.2)
Planned pregnancy	189 (27.8)	66 691 (82.0)
Missing	<5	115 (0.1)
Folic acid supplementation^c^	279 (41.1)	32 190 (39.6)
Illicit drug used^d^	33 (4.9)	468 (0.6)
Lifetime history of major depression	134 (19.7)	4672 (5.7)
Missing	20 (2.9)	1992 (2.4)
Sleeping problems	293 (43.2)	12 721 (15.6)
Anxiety	185 (27.2)	2710 (3.3)
Depression	211 (31.1)	5328 (6.5)
Adverse life event		
No	154 (22.7)	32 843 (40.4)
≥1, not painful	138 (20.3)	20 139 (24.8)
≥1, painful or very painful	387 (57.0)	28 377 (34.9)
Comedications anytime during pregnancy		
Nonsteroidal anti-inflammatory	98 (14.4)	5027 (6.2)
Opioids	97 (14.3)	1637 (2.0)
Paracetamol	443 (65.2)	36 666 (45.1)
Antidepressants	110 (16.2)	781 (1.0)
Antipsychotics	43 (6.3)	623 (0.8)
Antiepileptics	12 (1.8)	264 (0.3)
Depressive or anxiety symptoms during pregnancy, *z* score (SD)		
SCL-5 at week 17	0.89 (1.7)	−0.01 (0.99)
Missing	32 (4.7)	2589 (3.2)
SCL-8 at week 30	0.97 (1.7)	−0.01 (0.99)
Missing	60 (8.8)	6160 (7.6)

Abbreviations: BMI, body mass index (calculated as weight in kilograms divided by height in meters squared); SCL-5, the Hopkins Symptoms Checklist 5; SCL-8, the Hopkins Symptoms Checklist 8; z-hypnotics, benzodiazepine-like hypnotic drugs.

^a^ Highest level of either completed or ongoing education.

^b^ No or minimal alcohol intake indicates less than 1 alcoholic drink per month; low to moderate alcohol intake, 1 alcoholic drink per month to 1 alcoholic drink per week; frequent alcohol intake, more than 1 alcoholic drink per week.

^c^ Folic acid supplementation in the 4 weeks before pregnancy or up to week 12 of pregnancy.

^d^ Illicit drug use during pregnancy or the last month before pregnancy.

**Table 2.** Absolute Risks and Crude Outcome Distributions in Children Stratified by Exposure to Benzodiazepine or Benzodiazepine-like Hypnotic Drugs in Pregnancy

Outcome	No. (%)	
	Any exposure (n = 679)	No exposure (n = 81 359)
Gestational age, mean (SD), d	277 (13)	280 (12)
Preterm delivery	42 (6.1)	3480 (4.2)
Birth weight, mean (SD), g	3506 (592)	3613 (538)
Birth weight relative to gestational age and sex, mean (SD), *z* score	0.09 (0.95)	0.17 (1.08)
Small for gestational age	4 (6.6)	5029 (5.0)
Head circumference, mean (SD), cm	35.2 (1.9)	35.3 (1.6)
Apgar score <7 at 5 min	7 (1.0)	771 (0.9)
Respiratory distress	4 (0.6)	502 (0.6)

### Gestational Age and Preterm Delivery

All unadjusted and adjusted estimates are shown in Table 3. In the crude data, children born to mothers exposed to benzodiazepines or z-hypnotics were born at a mean (SD) gestational age of 277 (13) days, and had an absolute risk of 6.1% of preterm delivery. Children born to mothers without exposure had a mean (SD) gestational age of 280 (12) days and a 4.2% absolute risk of preterm delivery. The fully adjusted estimate in the full study population suggests that benzodiazepine or z-hypnotic use was associated with a lower gestational age at delivery by a mean difference of −2.1 (95% CI, −3.3 to −0.9) days. When considering timing of exposure, our estimates suggest that the association was stronger in midpregnancy (mean difference, −1.8 [95% CI, −4.5 to 1.0] days) and in late pregnancy (mean difference, −3.1 [95% CI, −5.7 to −0.5] days) compared with exposure in early pregnancy, (mean difference, 0.3 [95% CI, −1.0 to 1.7] days). There was no evidence of a cumulative dose-response association. In the analysis that examined preterm delivery as a binary outcome, any use of benzodiazepines or z-hypnotics was associated with an adjusted risk ratio (aRR) of 1.41 (95% CI, 1.03 to 1.94), with an aRR of 0.64 (95% CI, 0.36 to 1.12) for early pregnancy exposure, 1.33 (95% CI, 0.69 to 2.55) for mid-pregnancy exposure, and 2.17 (95% CI, 1.23 to 3.83) for late pregnancy exposure.

**Table 3.** Estimates of Effect of Exposure to Benzodiazepines or Benzodiazepine-like Hypnotic Drugs

Exposure	Relative risk (95% CI)		
	Crude analysis	Adjusted for baseline confounders^a^	Adjusted for baseline and time varying confounders^b^
**Gestational age, d**			
Never	0 [Reference]	0 [Reference]	0 [Reference]
Ever	−2.5 (−3.4 to −1.6)	−2.3 (−3.4 to −1.3)	−2.1 (−3.3 to −0.9)
Timing			
Unexposed	0 [Reference]	0 [Reference]	0 [Reference]
Early	0.1 (−1.1 to 1.2)	0.4 (−0.8 to 1.6)	0.4 (−1.0 to 1.7)
Mid	−2.0 (−3.6 to −0.3)	−2.3 (−4.6 to −0.1)	−1.8 (−4.5 to 1.0)
Late	−3.7 (−5.4 to −2.0)	−3.4 (−5.7 to −1.0)	−3.1 (−5.7 to −0.5)
Duration, 4-wk inteval			
0	0 [Reference]	0 [Reference]	0 [Reference]
1	−2.5 (−3.6 to −1.4)	−2.5 (−3.8 to −1.1)	−2.6 (−4.0 to −1.1)
2	−2.0 (−4.3 to 0.3)	−1.5 (−4.5 to 1.4)	−0.7 (−3.9 to 2.4)
≥3	−2.8 (−4.7 to −1.0)	−2.5 (−4.4 to −0.5)	−1.8 (−4.2 to 0.6)
**Preterm delivery**			
Never	1 [Reference]	1 [Reference]	1 [Reference]
Ever	1.55 (1.17 to 2.06)	1.45 (1.08 to 1.94)	1.41 (1.03 to 1.94)
Timing			
Unexposed	1 [Reference]	1 [Reference]	1 [Reference]
Early	0.80 (0.50 to 1.29)	0.71 (0.43 to 1.18)	0.64 (0.36 to 1.12)
Mid	1.10 (0.62 to 1.94)	1.18 (0.66 to 2.11)	1.33 (0.69 to 2.55)
Late	2.45 (1.55 to 3.86)	2.31 (1.38 to 3.86)	2.17 (1.23 to 3.83)
Duration, 4-wk inteval			
0	1 [Reference]	1 [Reference]	1 [Reference]
1	1.56 (1.09 to 2.22)	1.50 (1.05 to 2.14)	1.49 (1.03 to 2.16)
2	1.91 (0.98 to 3.71)	1.70 (0.87 to 3.36)	1.50 (0.73 to 3.09)
≥3	1.28 (0.65 to 2.51)	1.13 (0.56 to 2.26)	1.14 (0.51 to 2.57)
**Birth weight, g**			
Never	0 [Reference]	0 [Reference]	0 [Reference]
Ever	−106.2 (−147.0 to −65.4)	−78.5 (−123.0 to −34.0)	−79.3 (−126.7 to −31.9)
Timing			
Unexposed	0 [Reference]	0 [Reference]	0 [Reference]
Early	−25.5 (−79.8 to 28.8)	27.1 (−26.6 to 80.8)	25.8 (−34.2 to 85.8)
Mid	−109.1 (−186.1 to −32.1)	−107.8 (−198.7 to −16.9)	−82.2 (−190.8 to 26.4)
Late	−97.5 (−174.7 to −20.3)	−116.7 (−208.4 to −25.0)	−107.2 (−211.1 to −3.3)
Duration, 4-wk inteval			
0	0 [Reference]	0 [Reference]	0 [Reference]
1	−84.8 (−135.6 to −34.0)	−63.7 (−120.9 to −6.5)	−81.7 (−143.0 to −20.4)
2	−140.9 (−247.5 to −34.3)	−92.2 (−206.3 to 21.9)	−91.3 (−211.8 to 29.2)
≥3	−146.7 (−234.1 to −59.3)	−113.8 (−197.9 to −29.7)	−41.5 (−154.4 to 71.4)
**Birth weight relative to gestational age and sex, *z* score **			
Never	0 [Reference]	0 [Reference]	0 [Reference]
Ever	−0.08 (−0.16 to 0.00)	−0.04 (−0.12 to 0.04)	−0.04 (−0.12 to 0.04)
Timing			
Unexposed	0 [Reference]	0 [Reference]	0 [Reference]
Early	−0.08 (−0.20 to 0.04)	0.02 (−0.08 to 0.12)	0.01 (−0.09 to 0.11)
Mid	−0.12 (−0.28 to 0.04)	−0.10 (−0.24 to 0.04)	−0.05 (−0.21 to 0.11)
Late	0.02 (−0.14 to 0.18)	−0.05 (−0.19 to 0.09)	−0.06 (−0.22 to 0.10)
Duration, 4-wk inteval			
0	0 [Reference]	0 [Reference]	0 [Reference]
1	−0.03 (−0.15 to 0.09)	0.00 (−0.10 to 0.10)	−0.03 (−0.13 to 0.07)
2	−0.23 (−0.45 to −0.01)	−0.10 (−0.26 to 0.06)	−0.14 (−0.30 to 0.02)
≥3	−0.14 (−0.34 to 0.06)	−0.14 (−0.3 to 0.02)	−0.01 (−0.21 to 0.19)
**Small for gestational age**			
Never	1 [Reference]	1 [Reference]	1 [Reference]
Ever	1.24 (0.93 to 1.66)	1.11 (0.83 to 1.48)	0.96 (0.69 to 1.33)
Timing			
Unexposed	1 [Reference]	1 [Reference]	1 [Reference]
Early	1.15 (0.78 to 1.70)	0.93 (0.63 to 1.37)	0.87 (0.57 to 1.32)
Mid	1.39 (0.83 to 2.30)	1.35 (0.81 to 2.24)	0.98 (0.56 to 1.73)
Late	1.09 (0.63 to 1.88)	1.23 (0.74 to 2.04)	1.26 (0.69 to 2.33)
Duration, 4-wk inteval			
0	1 [Reference]	1 [Reference]	1 [Reference]
1	1.01 (0.68 to 1.51)	0.92 (0.62 to 1.37)	0.91 (0.6 to 1.38)
2	1.17 (0.54 to 2.55)	1.02 (0.46 to 2.27)	1.03 (0.45 to 2.39)
≥3	1.97 (1.22 to 3.19)	1.05 (0.65 to 1.71)	1.02 (0.54 to 1.91)
**Head circumference, cm**			
Never	0 [Reference]	0 [Reference]	0 [Reference]
Ever	−0.2 (−0.3 to −0.1)	−0.1 (−0.3 to 0.0)	−0.1 (−0.3 to 0.1)
Timing			
Unexposed	0 [Reference]	0 [Reference]	0 [Reference]
Early	−0.1 (−0.2 to 0.1)	0.1 (−0.1 to 0.3)	0.1 (−0.1 to 0.3)
Mid	−0.1 (−0.2 to 0.1)	0.0 (−0.4 to 0.3)	0.1 (−0.3 to 0.6)
Late	−0.4 (0.3 to −1.0)	−0.4 (−0.7 to −0.1)	−0.4 (−0.7 to −0.1)
Duration, 4-wk inteval			
0	0 [Reference]	0 [Reference]	0 [Reference]
1	−0.2 (−0.3 to 0)	−0.1 (−0.3 to 0.1)	−0.1 (−0.3 to 0.1)
2	−0.2 (−0.5 to 0.2)	0.0 (−0.5 to 0.5)	0.0 (−0.5 to 0.4)
≥3	−0.3 (−0.6 to −0.1)	−0.2 (−0.4 to 0.1)	0.1 (−0.3 to 0.4)
**Apgar Score <7 at 5 min**^**c**^			
Never	1 [Reference]	1 [Reference]	1 [Reference]
Ever	1.09 (0.52 to 2.28)	1.08 (0.51 to 2.29)	1.28 (0.59 to 2.79)
Timing			
Unexposed	1 [Reference]	1 [Reference]	1 [Reference]
Early	0.76 (0.23 to 2.48)	0.71 (0.20 to 2.58)	0.59 (0.15 to 2.28)
Mid	0.36 (0.04 to 2.91)	0.37 (0.04 to 3.86)	0.44 (0.03 to 5.57)
Late	2.01 (0.61 to 6.66)	2.06 (0.54 to 7.84)	2.90 (0.68 to 12.39)
**Respiratory distress**			
Never	1 [Reference]	1 [Reference]	1 [Reference]
Ever	0.96 (0.36 to 2.54)	0.94 (0.35 to 2.51)	0.61 (0.21 to 1.73)
Timing			
Unexposed	1 [Reference]	1 [Reference]	1 [Reference]
Early	0.89 (0.25 to 3.11)	0.90 (0.36 to 2.25)	0.69 (0.28 to 1.73)
Mid	2.13 (0.56 to 8.13)	2.11 (0.71 to 6.28)	1.48 (0.57 to 3.80)
Late	1.02 (0.21 to 5.06)	0.98 (0.18 to 5.41)	1.01 (0.26 to 3.94)
Duration, 4-wk inteval			
0	1 [Reference]	1 [Reference]	1 [Reference]
1	0.37 (0.05 to 2.64)	0.37 (0.05 to 2.62)	0.20 (0.03 to 1.45)
2	3.31 (0.84 to 13.08)	3.31 (0.80 to 13.71)	1.77 (0.42 to 7.50)
≥3	1.11 (0.16 to 7.84)	1.04 (0.14 to 7.65)	1.04 (0.14 to 7.68)

^a^ Baseline covariates: body mass index before conception, smoking, illicit drug use, alcohol intake, planned pregnancy, income, ongoing or completed education, adverse life events, sleeping and mental health issues, anxiety, and lifetime history of major depression.

^b^ Time-varying covariates: maternal symptoms of depression and anxiety during pregnancy, comedication use during pregnancy (ie, nonsteroidal anti-inflammatory drugs, opioids, paracetamol, antidepressants, antipsychotics, and antiepileptics), and fever during pregnancy.

^c^ For the outcome Apgar score less than 7 at 5 minutes, duration analysis is omitted as there were no events in some exposure groups.

### Birth Weight, Birth Weight Relative to Gestational Age and Sex, and Small for Gestational Age

The mean (SD) birth weight in children with benzodiazepine or z-hypnotic exposure was 3506 (592) g compared with 3613 (538) g in children without exposure. The crude absolute risk of being small for gestational age was 6.6% in the exposed group and in 5.0% in the unexposed group. The fully adjusted estimate suggests that exposure to benzodiazepines or z-hypnotics was associated with lower birth weight (mean difference, −79.3 [95% CI, −126.7 to −31.9] g). This strongest association was found in midpregnancy (mean difference, −82.2 [95% CI, −190.8 to 26.4] g) and late pregnancy (mean difference, −107.2 [95% CI, −211.1 to −3.3] g) rather than early pregnancy (mean difference, 25.8 [95% CI, −34.18 to 85.78] g). The adjusted analysis found no significant evidence of an association with birth weight relative to gestational age and sex (*z* scores) (mean difference, −0.04 [95% CI, −0.12 to 0.04]) or the binary variable small for gestational age (aRR, 0.96 [95% CI, 0.69 to 1.33]).

### Other Immediate Birth Outcomes

We did not find any significant evidence of an association of exposure to benzodiazepines or z-hypnotics with head circumference (mean difference, −0.07 [95% CI, −0.25 to 0.1] cm). Additionally, there was no statistically significant difference in Apgar score less than 7 at 5 minutes (aRR, 1.28 [95% CI, 0.59 to 2.79]) or risk of respiratory distress (aRR, 0.61 [95% CI, 0.21 to 1.73]).

### Subgroup Analyses and Sensitivity Analyses

eTable 1 and eTable 2 in the Supplement show the distribution of covariates between exposure groups in the disease sample and the benzodiazepine sample. The results from the disease sample were not significantly different from the results in the full study sample (eTable 3 in the Supplement). The results from the benzodiazepine sample, which was restricted to women who used benzodiazepines or z-hypnotics before pregnancy, shows a significant attenuation of the patterns from the primary analysis; in isolation, no comparisons from this analysis would be interpreted as being indicative of evidence for an association (eTable 4 in the Supplement).

In the analysis that restricted the exposure definition to benzodiazepines, to z-hypnotics, or to benzodiazepine-anxiolytics only (eTable 5 in the Supplement), the findings were generally consistent with the primary analysis, with differences that may be expected owing to sampling variability. We found no significant evidence of effect modification by sex of the child (eTable 6 in the Supplement). The analysis restricted to the first pregnancy per woman did not differ significantly from the primary analysis (eTable 7 in the Supplement).

## Discussion

This cohort study found that among children born to mothers participating in the MoBa cohort study, exposure to benzodiazepines or z-hypnotics during pregnancy was associated with slightly lower gestational age at delivery, slightly lower birth weight, and a slightly to moderately higher risk of preterm delivery. The timing analysis suggests that these results were primarily driven by exposure in the second and third trimester. We found no association with the child’s birth weight relative to gestational age and sex (*z* score) or any other immediate birth outcome.

One plausible interpretation of this pattern may be that benzodiazepine or z-hypnotic exposure (particularly late in pregnancy) results in earlier birth, and that this explains the lower birth weight in infants who were exposed to benzodiazepines or z-hypnotics in utero. In other words, the results are consistent with a hypothesis that these medications are not associated with impaired intrauterine grown but are associated with birth weight, primarily due to an association with pregnancy duration.

Our results for the association of benzodiazepines or z-hypnotics exposure with preterm delivery are consistent with a study by Wikner and colleagues^11^ that reported a nearly 50% increase in the odds of preterm delivery after use of benzodiazepines in early pregnancy, and a more than 150% increase in odds after use in late pregnancy. This association is much weaker than that reported by Calderon-Margalit et al,^12^ who reported a nearly 7-fold increase in odds of preterm delivery after use of benzodiazepines at any time during pregnancy. Earlier studies by Ornoy et al^10^ and Ogawa et al^9^ have not reported any adverse associations with birth weight.

In-utero exposure to benzodiazepines or z-hypnotics was much less common in our study than what has been observed in other countries but relatively similar to what has been observed in prescription databases in Norway. Norwegian prescription practices and cultural factors may explain the differences in use.

### Limitations

This study has some limitations. Observational studies are, by their nature, limited in their ability to establish causation with certainty. Consequently, our results are not necessarily reflective of a causal relationship. However, in the following discussion, it will be necessary to occasionally refer explicitly^25^ to the causal hypothesis as one possible explanation for the findings to discuss those limitations of our study that relate specifically to the question of causality.

Residual confounding cannot be fully ruled out as an explanation for these findings. While we controlled for a binary covariate for baseline anxiety and a time-dependent Hopkins Symptoms Checklist that includes a component for anxiety during pregnancy, these constructs may not be able to fully capture and control the confounding effects of anxiety, which is the primary indication for benzodiazepines and z-hypnotics and which is also known to reduce gestational duration.^26^ In this context, it may be worth noting that the association was primarily seen in the second and third trimester. If symptoms of anxiety do not vary significantly during the course of pregnancy, and if the findings were explained by confounding by unmeasured aspects of anxiety, one would expect to see just as strong an association in the first trimester.

When the analysis is restricted to the benzodiazepine sample, which included only women who used benzodiazepines or z-hypnotics before pregnancy, the data no longer provide any clear indication of an association with continued use. The benzodiazepine sample was subject to less confounding by indication than the full study sample (because all women in the benzodiazepine sample had sufficiently strong indications for benzodiazepine use that the medications were used shortly before pregnancy). Therefore, it is not entirely implausible that the attenuated results from the benzodiazepine sample represent a more accurate estimate of the true association. However, the benzodiazepine sample was very small and potentially underpowered, which could prevent detection of a true association.

While we hypothesize that the estimated association of benzodiazepines or z-hypnotics with birth weight was primarily driven by the effect of exposure on gestational duration, we were not able to test this hypothesis formally in a mediation analysis. Such an analysis was not planned or prespecified and would also have been challenging given potential for strong confounding of the mediator-outcome association. If mediator-outcome confounding can be addressed adequately, such mediation analysis may be a target for future work.

The analysis for cumulative duration of exposure did not show evidence of a dose-response trend. However, this type of analysis may not capture the true dose-response association, as we only had access to data on number of exposure intervals during which use of medications were reported. The cumulative exposure within each exposure window may differ substantially between women.

Our study is based on self-reported exposure; therefore, underreporting of medication use is a potential limitation. However, self-reported exposure in MoBa has been shown to correlate well with drug prescription data.^27^ The self-selected participants in MoBa have been shown to differ from women in MBRN who did not enroll in MoBa in terms of several baseline characteristics, including age and social status. While this may be a threat to generalizability and external validity, earlier analysis using data from MBRN has shown that for several exposures and for several immediate birth outcomes, the measures of association did not differ between women enrolled in MoBa and women not enrolled in MoBa.^28^

## Conclusions

This cohort study found that children born to mothers who were exposed to benzodiazepines or z-hypnotics in pregnancy had slightly lower birth weight, were born at approximately 2 days younger gestational age, and had slightly higher to moderately higher risk of preterm birth compared with children without exposure. These patterns were only slightly attenuated when controlling for baseline and time-dependent covariates and were primarily associated with exposure in middle and late pregnancy. The lower birth weight in children exposed to benzodiazepines or z-hypnotics was not necessarily due to impaired intrauterine growth and could potentially be explained by earlier delivery. While the magnitudes of the associations were not necessarily clinically significant, benzodiazepines and z-hypnotics are not first-line treatment for either anxiety or insomnia and should only be used in pregnancy after a thorough evaluation of the benefits and risks for the mother and child.

## Notes

Supplement. **eAppendix 1.** Missing Data **eAppendix 2.** Statistical Models **eReferences** **eTable 1.** Maternal and Paternal Characteristics Stratified by Maternal Exposure to Benzodiazepines or Benzodiazepine-like Hypnotic Drugs During Pregnancy (Disease Sample) **eTable 2.** Maternal and Paternal Characteristics Stratified by Maternal Exposure to Benzodiazepines or Benzodiazepine-like Hypnotic Drugs During Pregnancy (Benzodiazepine Sample) **eTable 3.** Unadjusted and Adjusted Estimates of Effect of Exposure to Benzodiazepines or Benzodiazepine-like Hypnotic Drugs Unadjusted Analyses in the Disease Sample **eTable 4.** Unadjusted and Adjusted Estimates of Effect of Exposure to Benzodiazepines or Benzodiazepine-like Hypnotic Drugs in the Benzodiazepines Sample **eTable 5.** Effect Estimates With Exposure Definition Restricted to Benzodiazepines Only, Benzodiazepine-like Hypnotic Drugs only, or Benzodiazepine Anxiolytics Only **eTable 6.** Effects Estimates For Exposure To Benzodiazepines Or Benzodiazepine-like Hypnotic Drugs Stratified by Sex of Offspring **eTable 7.** Effects Estimates for Exposure to Benzodiazepines or Benzodiazepine-like Hypnotic Drugs in Dataset Restricted to First-Time Participants in the Norwegian Mother, Father and Child Cohort Study
