# Supplementary material for: Associations of Maternal Use of Benzodiazepines or Benzodiazepine-like Hypnotics During Pregnancy With Immediate Pregnancy Outcomes in Norway
Source: JAMA Netw Open. 2020 Jun 22;3(6):e205860. doi: 10.1001/jamanetworkopen.2020.5860 (PMC7309438; doi:10.1001/jamanetworkopen.2020.5860)
Supplement: Supplement. — eAppendix 1. Missing Data eAppendix 2. Statistical Models eReferences eTable 1. Maternal and Paternal Characteristics Stratified by Maternal Exposure to Benzodiazepines or Benzodiazepine-like Hypnotic Drugs During Pregnancy (Disease Sample) eTable 2. Maternal and Paternal Characteristics Stratified by Maternal Exposure to Benzodiazepines or Benzodiazepine-like Hypnotic Drugs During Pregnancy (Benzodiazepine Sample) eTable 3. Unadjusted and Adjusted Estimates of Effect of Exposure to Benzodiazepines or Benzodiazepine-like Hypnotic Drugs Unadjusted Analyses in the Disease Sample eTable 4. Unadjusted and Adjusted Estimates of Effect of Exposure to Benzodiazepines or Benzodiazepine-like Hypnotic Drugs in the Benzodiazepines Sample eTable 5. Effect Estimates With Exposure Definition Restricted to Benzodiazepines Only, Benzodiazepine-like Hypnotic Drugs only, or Benzodiazepine Anxiolytics Only eTable 6. Effects Estimates For Exposure To Benzodiazepines Or Benzodiazepine-like Hypnotic Drugs Stratified by Sex of Offspring eTable 7. Effects Estimates for Exposure to Benzodiazepines or Benzodiazepine-like Hypnotic Drugs in Dataset Restricted to First-Time Participants in the Norwegian Mother, Father and Child Cohort Study [file jamanetwopen-3-e205860-s001.pdf]

Supplementary Online Content

Huitfeldt A, Sundbakk LM, Skurtveit S, Handal M, Nordeng H. Associations of maternal use of benzodiazepines and benzodiazepine-like hypnotic drugs during pregnancy with immediate pregnancy outcomes in Norway. *JAMA Netw Open*. 2020;3(6):e205860. doi:10.1001/jamanetworkopen.2020.5860

eAppendix 1. Missing Data

eAppendix 2. Statistical Models

eReferences

eTable 1. Maternal and Paternal Characteristics Stratified by Maternal Exposure to Benzodiazepines or Benzodiazepine-like Hypnotic Drugs During Pregnancy (Disease Sample)

eTable 2. Maternal and Paternal Characteristics Stratified by Maternal Exposure to Benzodiazepines or Benzodiazepine-like Hypnotic Drugs During Pregnancy (Benzodiazepine Sample)

eTable 3. Unadjusted and Adjusted Estimates of Effect of Exposure to Benzodiazepines or Benzodiazepine-like Hypnotic Drugs Unadjusted Analyses in the Disease Sample

eTable 4. Unadjusted and Adjusted Estimates of Effect of Exposure to Benzodiazepines Benzodiazepine-like Hypnotic Drugs in the Benzodiazepines Sample

eTable 5. Effect Estimates With Exposure Definition Restricted to Benzodiazepines Only, Benzodiazepine-like Hypnotic Drugs only, or Benzodiazepine Anxiolytics Only

eTable 6. Effects Estimates For Exposure To Benzodiazepines Or Benzodiazepine-like Hypnotic Drugs Stratified by Sex of Offspring

eTable 7. Effects Estimates for Exposure to Benzodiazepines or Benzodiazepine-like Hypnotic Drugs in Dataset Restricted to First-Time Participants in the Norwegian Mother, Father and Child Cohort Study

This supplementary material has been provided by the authors to give readers additional information about their work.

© 2020 Huitfeldt A et al. *JAMA Network Open.*

eAppendix 1. Missing Data

There are two separate missing data mechanisms that arise when using data obtained from linking MoBa with MBRN. The first mechanism is that some women enroll in MoBa but fail to return the questionnaires, leading to large blocks of missing data across many variables. The second mechanism is that some individual variables are missing in some observations, for example due to non-response on a MoBa question or individually missing data in MBRN. We used a combination of inverse probability of censoring (IPC) weights and multiple imputation, to account for both these types of missingness:

1. In step one, we used logistic regression models to predict the probability of being censored due to failure to complete MoBa Q1, Q3, and Q4; conditional on fully observed past covariates. The model for failure to return Q1 is fit in the expanded dataset of 109 827 singleton pregnancies (with parameters for age, marriage status, parity, sex of offspring, and health region), the model for failure to return Q3 is fit in those who completed Q1 (and adds the following variables from Q1: pre-pregnancy and early pregnancy exposure to BZDs/z-hypnotics, paracetamol, NSAIDS, antidepressants antipsychotics and opioids), and the model for failure to return Q4 is fit in those who completed Q3 and Q4 (and adds the following variables from Q3: mid-pregnancy exposure to BZDs/z-hypnotics, paracetamol, NSAIDS, antidepressants antipsychotics and opioids). For every observation, the probability of censoring is the computed as the product of the predicted probability of censoring at Q1, Q3 and Q4.
2. In step two (and in all subsequent analyses), we restricted our dataset to the 82 038 observations in which the mother completed all three questionnaires. Individually-missing covariates were imputed using multiple imputation by chained equations (R package MICE(1)). We used 20 iterations to create 20 imputed datasets. All covariates used in the statistical analysis were also used in the imputation models. The covariates with individually missing data were: Pre-pregnancy BMI (1.9%), maternal education (0.4%), smoking (1.3%), alcohol intake (8.2%), income (3.2%), planned pregnancy (0.1%), LTH of MD (2.4%), depressive/anxiety symptoms during pregnancy (3.2% on Q1, 7.6% on Q3).
3. In step 3, the statistical analysis was conducted separately in each imputed dataset. All observations in this analysis are weighted by their inverse of the probability of not being censored by failure to complete questionnaires, as estimated in step 1. The effect estimates from each imputed dataset were then combined across imputed datasets(2) to obtain an overall estimate (R package Amelia(3)).

   The IPC weights used to account for missing data are separate from the IPT weights used to account for time-varying confounding; in the final analysis the observations are weighted by the product of the two.

© 2020 Huitfeldt A et al. *JAMA Network Open.*

eAppendix 2. Statistical Models

To account both for time-fixed and time-varying confounders, we estimated treatment effects using marginal structural models. Linear models were used for continuous outcomes (birth weight, z-scores, gestational age at birth and head circumference), and log-binomial models were used for binary outcomes (small for gestational age, low Apgar score at 5 minutes, respiratory distress). These models included parameters for the intervention variables (BZDs/z-hypnotics) and for all baseline confounders discussed above. For all outcomes, we fit three separate outcome models that used different functional forms for the intervention variables: Effect of ever use vs never use (indicator for ever use during pregnancy), effect of timing (indicator variables for early pregnancy use, mid-pregnancy use and late pregnancy use of BZDs/z-hypnotics), and effect of duration of exposure (indicator variables for use of BZDs/z-hypnotics in 1 interval, use in 2 intervals, and use in 3+ intervals during pregnancy). Robust variance estimators were used to estimate standard errors.

In order to control for confounding by time-varying covariates, all observations were weighted by stabilized inverse probability of treatment (IPT) weights. Because our outcome variable is measured at the same time for all records in the dataset (that is, at birth), these weights are time-fixed. For the IPT weights, we estimated the probability of BZD/z-hypnotic treatment using logistic regression models (separately for early pregnancy exposure, mid-pregnancy exposure and late pregnancy exposure) conditional on time-fixed confounders, the history of the time-varying confounders up until the current time point, and the history of BZD/z-hypnotic history treatment up until the current time point. For each pregnancy, the denominator of the IPT weights was then computed as the product of the predicted probability of the observed treatment across all time points. In those outcome models where we are considering ever use vs never use, we set the contributions to the weights to 1 for all time periods following first exposure. The numerator of the stabilized weights was computed in a similar fashion, but time-dependent covariates were not included in the models for exposure.

© 2020 Huitfeldt A et al. *JAMA Network Open.*

eReferences

1. Buuren S van, Groothuis-Oudshoorn K. mice: Multivariate Imputation by Chained Equations in R. J Stat Softw. 2011;45(3):1–67.

2. Leyrat C, Seaman SR, White IR, Douglas I, Smeeth L, Kim J, et al. Propensity score analysis with partially observed covariates: How should multiple imputation be used? Stat Methods Med Res. 2019 Jan;28(1):3–19.

3. Honaker J, King G, Blackwell M. Amelia II: A Program for Missing Data. J Stat Softw. 2011;45(7):1–47.

© 2020 Huitfeldt A et al. *JAMA Network Open.*

eTable 1. Maternal and Paternal Characteristics Stratified by Maternal Exposure to Benzodiazepines or Benzodiazepine-like Hypnotic Drugs During Pregnancy (Disease Sample)

	**BZD/z-hypnotic exposure during pregnancy**		
**Variable**	**Yes (n=465)**		**No (n=19089)**
Age in years, mean ± SD	31.2 ± 4.7		30.3 ± 4.8
Married/cohabiting, No (%)	412 (88.6)		18089 (94.8)
Primiparous, No (%)	220 (47.3)		9222 (48.3)
Pre-pregnancy BMI, kg/m^2; mean ± SD	24.1 ± 4.4		24.1 ± 4.4
Missing, No (%)	9 (1.9)		521 (2.7)
College/university education, No (%)	184 (39.6)		7452 (39.0)
Missing, No (%)	<5		89 (0.5)
Smoking, No (%)	82 (17.6)		1810 (9.5)
Missing, No (%)	<5		184 (1.0)
Alcohol intake during pregnancy, No (%)			
No or minimal	396 (85.2)		16982 (89.0)
Low to moderate	22 (4.7)		500 (2.6)
Frequent	<5		23 (0.1)
Missing, No (%)	46 (9.9)		1584 (8.3)
Gross yearly income, No (%)			
Average	353 (75.9)		14162 (74.2)
Low	46 (9.9)		1981 (10.4)
High	48 (10.3)		2354 (12.3)
Missing, No (%)	18 (3.9)		592 (3.1)
Planned pregnancy, No (%)	130 (28)		14914 (78.1)
Missing, No (%)	<5		32 (0.2)
Folic acid supplementation, No (%)	193 (41.5)		7496 (39.3)
Illicit drug used, No (%)	25 (5.4)		207 (1.1)
LTH of MD, No (%)	120 (25.8)		2659 (13.9)
Missing, No (%)	14 (3.0)		568 (3.0)
Sleeping problems, No (%)	293 (63.0)		12721 (66.6)
Anxiety, No (%)	185 (39.8)		2710 (14.2)
Depression, No (%)	211 (45.4)		5328 (27.9)
Adverse life event, No (%)			
No	73 (15.7)		5231 (27.4)
At least one, not painful	97 (20.9)		4780 (25.0)
At least one, painful/very painful	295 (63.4)		9078 (47.6)
Comedications anytime during pregnancy, No (%)			
NSAIDs	72 (15.5)		1513 (7.9)
Opioids	63 (13.5)		577 (3.0)
Paracetamol	303 (65.2)		9668 (50.6)
Antidepressants	108 (23.2)		740 (3.9)
Antipsychotics	39 (8.4)		243 (1.3)
Antiepileptics	10 (2.2)		90 (0.5)
Depressive/anxiety symptoms during pregnancy, z-score ± sd			
SCL-5 at week 17	1.26 ± 1.82		0.55 ± 1.37
Missing, No (%)	23 (4.9)		760 (4.0)
SCL-8 at week 30	1.35 ± 1.86		0.55 ± 1.37
Missing, No (%)	41 (8.8)		1430 (7.5)

© 2020 Huitfeldt A et al. *JAMA Network Open.*

eTable 2. Maternal and Paternal Characteristics Stratified by Maternal Exposure to Benzodiazepines or Benzodiazepine-like Hypnotic Drugs During Pregnancy (Benzodiazepine Sample)

	**BZD/z-hypnotic exposure during pregnancy**	
**Variable**	**Yes (n=219)**	**No (n=425)**
Age in years, mean ± SD	31.3 ± 4.9	30 ± 5
Married/cohabiting, No (%)	189 (86.3)	379 (89.2)
Primiparous, No (%)	128 (58.4)	180 (42.4)
Pre-pregnancy BMI, kg/m^2; mean ± SD	23.2 ± 3.9	23.6 ± 4.2
Missing, No (%)	<5	9 (2.1)
College/university education, No (%)	87 (39.7)	184 (43.3)
Missing, No (%)	<5	<5
Smoking, No (%)	48 (21.9)	68 (16.0)
Missing, No (%)	<5	<5
Alcohol intake during pregnancy, No (%)		
No or minimal	183 (83.6)	341 (80.2)
Low to moderate	10 (4.6)	17 (4.0)
Frequent	<5	<5
Missing, No (%)	25 (11.4)	56 (13.2)
Gross yearly income, No (%)		
Average	154 (70.3)	300 (70.6)
Low	30 (13.7)	53 (12.5)
High	28 (12.8)	57 (13.4)
Missing, No (%)	7 (3.2)	<5
Planned pregnancy, No (%)	74 (33.8)	124 (29.2)
Missing, No (%)	<5	<5
Folic acid supplementation, No (%)	83 (37.9)	134 (31.5)
Illicit drug used, No (%)	20 (9.1)	22 (5.2)
LTH of MD, No (%)	57 (26.0)	83 (19.5)
Missing, No (%)	<5	<5
Sleeping problems, No (%)	97 (44.3)	129 (30.4)
Anxiety, No (%)	88 (40.2)	139 (32.7)
Depression, No (%)	91 (41.6)	164 (38.6)
Adverse life event, No (%)		
No	41 (18.7)	96 (22.6)
At least one, not painful	38 (17.4)	78 (18.4)
At least one, painful/very painful	140 (63.9)	241 (56.7)
Comedications anytime during pregnancy, No (%)		
NSAIDs	40 (18.3)	38 (8.9)
Opioids	33 (15.1)	23 (5.4)
Paracetamol	145 (66.2)	224 (52.7)
Antidepressants	51 (23.3)	39 (9.2)
Antipsychotics	16 (7.3)	11 (2.6)
Antiepileptics	7 (3.2)	5 (1.2)
Depressive/anxiety symptoms during pregnancy, z-score ± sd		
SCL-5 at week 17	1.33 ± 1.94	0.96 ± 1.7
Missing, No (%)	13 (5.9)	12 (2.8)
SCL-8 at week 30	1.27 ± 1.88	0.74 ± 1.55
Missing, No (%)	19 (8.7)	27 (6.4)

© 2020 Huitfeldt A et al. *JAMA Network Open.*

eTable 3. Unadjusted and Adjusted Estimates of Effect of Exposure to Benzodiazepines or Benzodiazepine-like Hypnotic Drugs Unadjusted Analyses in the Disease Sample

	Crude analysis	Adjusted for baseline confounders[1]	Adjusted for baseline and time-varying confounders[2]
**Gestational age (days)**			
Ever/Never			
Never	0 (Reference)	0 (Reference)	0 (Reference)
Ever	-2.3 (-3.4, -1.2)	-2.4 (-3.7, -1.1)	-2.2 (-3.6, -0.7)
Timing			
Unexposed	0 (Reference)	0 (Reference)	0 (Reference)
Early	-0.1 (-1.5, 1.4)	0.1 (-0.1, 0.3)	0.1 (-1.5, 1.7)
Mid	-2.1 (-4.2, 0.0)	-2.8 (2.6, -8.2)	-2.6 (-6.2, 1.0)
Late	-2.3 (-4.4, -0.2)	-1.9 (1.9, -5.7)	-1.3 (-4.6, 1.9)
Duration			
0	0 (Reference)	0 (Reference)	0 (Reference)
1	-2.4 (-3.9, -1.0)	-2.7 (-4.5, -0.9)	-2.9 (-4.8, -1.0)
2	-2.0 (-4.8, 0.8)	-1.8 (-5.4, 1.8)	-1.0 (-5.0, 3.1)
3+	-2.1 (-4.2, 0.0)	-2.1 (-4.1, 0.0)	-1.4 (-3.9, 1.1)
**Preterm delivery (RR)**			
Ever/Never			
Never	1 (Reference)	1 (Reference)	1 (Reference)
Ever	1.37 (0.96, 1.95)	1.37 (0.95, 1.96)	1.32 (0.90, 1.94)
Timing			
Unexposed	1 (Reference)	1 (Reference)	1 (Reference)
Early	0.86 (0.49, 1.48)	0.80 (0.44, 1.45)	0.71 (0.37, 1.35)
Mid	1.18 (0.59, 2.36)	1.32 (0.65, 2.70)	1.49 (0.68, 3.26)
Late	1.65 (0.87, 3.14)	1.59 (0.75, 3.36)	1.49 (0.66, 3.34)
Duration			
0	1 (Reference)	1 (Reference)	1 (Reference)
1	1.44 (0.92, 2.25)	1.48 (0.94, 2.31)	1.46 (0.91, 2.32)
2	1.79 (0.83, 3.87)	1.74 (0.79, 3.82)	1.59 (0.69, 3.69)
3+	1.00 (0.46, 2.20)	0.94 (0.42, 2.11)	0.90 (0.36, 2.23)
**Birth weight (grams)**			
Ever/Never			
Never	0 (Reference)	0 (Reference)	0 (Reference)
Ever	-103.1 (-153.3, -52.9)	-86.4 (-140, -32.7)	-90.0 (-147.3, -32.6)
Timing			
Unexposed	0 (Reference)	0 (Reference)	0 (Reference)
Early	-34.1 (-100.4, 32.2)	4.7 (-60.3, 69.7)	6.7 (-63.6, 77.0)
Mid	-103.9 (-199.4, -8.4)	-100.0 (-214.1, 14.1)	-89.2 (-225.4, 47.0)
Late	-47.3 (-144.4, 49.8)	-76.1 (-188.6, 36.5)	-46 (-174.6, 82.6)
Duration			
0	0 (Reference)	0 (Reference)	0 (Reference)
1	-96.3 (-162.3, -30.4)	-87.9 (-162.5, -13.4)	-105.8 (-183.7, -27.9)
2	-123.1 (-250.2, 4.0)	-84.5 (-219.9, 50.9)	-89.3 (-236.3, 57.7)
3+	-106.0 (-200.8, -11.2)	-84.2 (-169.9, 1.4)	-19.6 (-128.9, 89.7)
**Birth weight relative to gestational age and sex**			

© 2020 Huitfeldt A et al. *JAMA Network Open.*

**(z-scores)**			
Ever/Never			
Never	0 (Reference)	0 (Reference)	0 (Reference)
Ever	-0.08 (-0.17, 0.01)	-0.05 (-0.14, 0.03)	-0.04 (-0.13, 0.05)
Timing			
Unexposed	0 (Reference)	0 (Reference)	0 (Reference)
Early	-0.10 (-0.22, 0.01)	-0.03 (-0.14, 0.08)	-0.03 (-0.14, 0.09)
Mid	-0.06 (-0.23, 0.10)	-0.04 (-0.19, 0.10)	-0.01 (-0.17, 0.16)
Late	0.03 (-0.14, 0.20)	-0.04 (-0.20, 0.12)	-0.03 (-0.22, 0.17)
Duration			
0	0 (Reference)	0 (Reference)	0 (Reference)
1	-0.04 (-0.15, 0.06)	-0.03 (-0.14, 0.08)	-0.06 (-0.17, 0.06)
2	-0.09 (-0.31, 0.13)	-0.06 (-0.23, 0.12)	-0.09 (-0.27, 0.09)
3+	-0.14 (-0.31, 0.02)	-0.10 (-0.25, 0.05)	0.01 (-0.17, 0.18)
**Small for gestational age (RR)**			
Ever/Never			
Never	1 (Reference)	1 (Reference)	1 (Reference)
Ever	1.13 (0.78, 1.64)	1.01 (0.70, 1.47)	0.78 (0.52, 1.16)
Timing			
Unexposed	1 (Reference)	1 (Reference)	1 (Reference)
Early	1.48 (0.95, 2.30)	1.23 (0.80, 1.87)	1.15 (0.74, 1.80)
Mid	0.74 (0.35, 1.59)	0.72 (0.37, 1.40)	0.50 (0.24, 1.04)
Late	1.14 (0.56, 2.30)	1.25 (0.66, 2.35)	0.78 (0.40, 1.54)
Duration			
0	1 (Reference)	1 (Reference)	1 (Reference)
1	1.03 (0.61, 1.71)	0.95 (0.58, 1.57)	0.86 (0.51, 1.45)
2	0.82 (0.27, 2.50)	0.72 (0.23, 2.24)	0.73 (0.23, 2.29)
3+	1.52 (0.84, 2.77)	1.27 (0.69, 2.33)	0.64 (0.32, 1.28)
**Head circumference (cms)**			
Ever/Never			
Never	0 (Reference)	0 (Reference)	0 (Reference)
Ever	-0.2 (-0.4, -0.1)	-0.1 (-0.3, 0.1)	-0.1 (-0.3, 0.1)
Timing			
Unexposed	0 (Reference)	0 (Reference)	0 (Reference)
Early	0.0 (-0.2, 0.2)	0.1 (-0.1, 0.3)	0.1 (-0.1, 0.4)
Mid	-0.1 (-0.4, 0.2)	-0.1 (-0.4, 0.3)	0.1 (-0.5, 0.7)
Late	-0.3 (-0.6, 0.0)	-0.3 (-0.7, 0.1)	-0.4 (-0.8, 0.1)
Duration			
0	0 (Reference)	0 (Reference)	0 (Reference)
1	-0.2(-0.4, 0.0)	-0.1 (-0.4, 0.1)	-0.2 (-0.5, 0.2)
2	-0.4 (-0.8, -0.0)	-0.3 (-0.7, 0.1)	-0.3 (-0.7, 0.1)
3+	-0.2 (-0.4, 0.1)	-0.1 (-0.3, 0.2)	0.2 (-0.2, 0.6)
**Apgar score <7 at 5 minutes (RR)**[3]			
Ever/Never			
Never	1 (Reference)	1 (Reference)	1 (Reference)
Ever	0.94 (0.35, 2.53)	0.96 (0.35, 2.61)	1.29 (0.44, 3.79)
Timing			
Unexposed	1 (Reference)	1 (Reference)	1 (Reference)
Early	0.76 (0.17, 3.33)	0.76 (0.14, 4.10)	0.56 (0.09, 3.71)

© 2020 Huitfeldt A et al. *JAMA Network Open.*

Mid	0.75 (0.08, 6.78)	0.76 (0.05, 11.41)	0.70 (0.03, 16.89)
Late	0.85 (0.10, 7.40)	0.85 (0.06, 11.21)	2.10 (0.13, 34.41)
Duration			
**Respiratory distress (RR)**			
Ever/Never			
Never	1 (Reference)	1 (Reference)	1 (Reference)
Ever	1.27 (0.47, 3.43)	1.25 (0.46, 3.38)	1.06 (0.38, 2.97)
Timing			
Unexposed	1 (Reference)	1 (Reference)	1 (Reference)
Early	0.96 (0.25, 3.68)	0.97 (0.39, 2.44)	0.77 (0.2, 2.97)
Mid	2.50 (0.57, 11.00)	2.39 (0.68, 8.43)	1.70 (0.39, 7.47)
Late	1.67 (0.30, 9.50)	1.02 (0.13, 7.89)	1.13 (0.20, 6.40)
Duration			
0	1 (Reference)	1 (Reference)	1 (Reference)
1	0.56 (0.08, 3.97)	1.79 (0.25, 12.63)	0.30 (0.04, 2.15)
2	4.17 (1.05, 16.52)	4.34 (1.04, 18.11)	2.22 (0.51, 9.69)
3+	1.16 (0.16, 8.21)	1.06 (0.15, 7.59)	1.11 (0.15, 8.00)
**Small for gestational age (RR)**			
Ever/Never			
Never	1 (Reference)	1 (Reference)	1 (Reference)
Ever	1.13 (0.78, 1.64)	1.01 (0.70, 1.47)	0.78 (0.52, 1.16)
Timing			
Unexposed	1 (Reference)	1 (Reference)	1 (Reference)
Early	1.48 (0.95, 2.30)	1.23 (0.80, 1.87)	1.15 (0.74, 1.8)
Mid	0.74 (0.35, 1.59)	0.72 (0.37, 1.40)	0.50 (0.24, 1.04)
Late	1.14 (0.56, 2.30)	1.25 (0.66, 2.35)	0.78 (0.40, 1.54)
Duration			
0	1 (Reference)	1 (Reference)	1 (Reference)
1	1.03 (0.61, 1.71)	0.95 (0.58, 1.57)	0.86 (0.51, 1.45)
2	0.82 (0.27, 2.50)	0.72 (0.23, 2.24)	0.73 (0.23, 2.29)
3+	1.52 (0.84, 2.77)	1.27 (0.69, 2.33)	0.64 (0.32, 1.28)

[1]Baseline covariates: BMI before conception, smoking, illicit drug use, alcohol intake, planned pregnancy, income, ongoing or completed education, adverse life events, sleeping and mental health problems, anxiety and LTH of MD.

[2]Time-varying covariates: Maternal symptoms of depression and anxiety during pregnancy, comedication use during pregnancy (NSAIDS, opioids, Paracetamol, antidepressants, antipsychotics and antiepileptics) and fever during pregnancy.

[3]For the outcome Apgar score less than 7 at 5 minutes, duration analysis is omitted as there were no events in some exposure groups.

© 2020 Huitfeldt A et al. *JAMA Network Open.*

eTable 4. Unadjusted and Adjusted Estimates of Effect of Exposure to Benzodiazepines Benzodiazepine-like Hypnotic Drugs in the Benzodiazepines Sample.

	Crude analysis	Adjusted for baseline confounders[1]	Adjusted for baseline and time-varying confounders[2]
**Gestational age (days)**			
Discontinuer	0 (Reference)	0 (Reference)	0 (Reference)
Continuer	-0.2 (-2.1, 1.6)	-0.4 (-2.3, 1.5)	-0.3 (-2.2, 1.7)
**Birth weight (grams)**			
Discontinuer	0 (Reference)	0 (Reference)	0 (Reference)
Continuer	-41.4 (-123.5, 40.7)	-19.1 (-104.3, 66.1)	-18.2 (-105.2, 68.7)
**Birth weight relative to gestational age and sex (z-scores)**			
Discontinuer	0 (Reference)	0 (Reference)	0 (Reference)
Continuer	-0.08 (0.07, -0.23)	-0.02 (-0.18, 0.13)	0.06 (-0.13, 0.26)
**Head circumference (cms)**			
Discontinuer	0 (Reference)	0 (Reference)	0 (Reference)
Continuer	-0.1 (-0.4, 0.2)	0.0 (-0.3, 0.4)	0.0 (-0.3, 0.4)
**Apgar score less than 7 at 5 minutes (RR)**			
Discontinuer	1 (Reference)	1 (Reference)	1 (Reference)
Continuer	0.95 (0.17, 5.15)	0.97 (0.12, 8.02)	0.88 (0.10, 7.99)

[1]Baseline covariates: BMI before conception, smoking, illicit drug use, alcohol intake, planned pregnancy, income, ongoing or completed education, adverse life events, sleeping and mental health problems, anxiety and LTH of MD.

[2]Time-varying covariates: Maternal symptoms of depression and anxiety during pregnancy, comedication use during pregnancy (NSAIDS, opioids, Paracetamol, antidepressants, antipsychotics and antiepileptics) and fever during pregnancy.

Models for risk of respiratory distress, preterm delivery and small for gestational age did not converge

© 2020 Huitfeldt A et al. *JAMA Network Open.*

eTable 5. Effect Estimates With Exposure Definition Restricted to Benzodiazepines Only, Benzodiazepine-like Hypnotic Drugs only, or Benzodiazepine Anxiolytics Only

5a: Effect estimates with exposure definition restricted to BZDs (N05BA, N05CD and N03AE01) (n=82038, exposed: 461). 95% confidence intervals

	Crude analysis	Adjusted for baseline confounders[1]	Adjusted for baseline and time varying confounders[2]
**Gestational age (days)**			
Ever/Never			
Never	0 (Reference)	0 (Reference)	0 (Reference)
Ever	-3.3 (-4.4, -2.2)	-3.0 (-4.3, -1.6)	-3.1 (-4.8, -1.4)
Timing			
Unexposed	0 (Reference)	0 (Reference)	0 (Reference)
Early	0.0 (-1.2, 1.2)	0.4 (-0.7, 1.5)	0.1 (-1.2, 1.4)
Mid	-1.5 (-3.2, 0.2)	-1.9 (-4.1, 0.3)	-0.9 (-3.6, 1.7)
Late	-3.2 (-5.0, -1.5)	-2.9 (-5.2, -0.5)	-2.7 (-5.4, 0.0)
**Preterm delivery (RR)**			
Ever/Never			
Never	1 (Reference)	1 (Reference)	1 (Reference)
Ever	1.9 (1.39, 2.60)	1.75 (1.25, 2.44)	1.92 (1.32, 2.78)
Timing			
Unexposed	1 (Reference)	1 (Reference)	1 (Reference)
Early	0.76 (0.46, 1.27)	0.67 (0.39, 1.14)	0.64 (0.36, 1.13)
Mid	1.03 (0.56, 1.89)	1.13 (0.61, 2.07)	1.14 (0.56, 2.31)
Late	2.14 (0.66, 6.93)	2.01 (1.14, 3.56)	1.97 (1.05, 3.70)
**Birth weight (grams)**			
Ever/Never			
Never	0 (Reference)	0 (Reference)	0 (Reference)
Ever	-72.4 (-123.4, -21.3)	-58.11 (-116.2, 0.0)	-58.4 (-116.4, -0.3)
Timing			
Unexposed	0 (Reference)	0 (Reference)	0 (Reference)
Early	-32.0 (-87.5, 23.4)	21.8 (-32.1, 75.7)	14.6 (-44.9, 74)
Mid	-99.8 (-179.4, -20.1)	-96.7 (-187.04, -6.3)	-56.6 (-165.1, 51.8)
Late	-82.8 (-163.6, -1.9)	-99.44 (-192.7, -6.2)	-90.9 (-198.3, 16.5)
**Birth weight relative to gestational age and sex (z-scores)**			
Ever/Never			
Never	0 (Reference)	0 (Reference)	0 (Reference)
Ever	0.03 (-0.07, 0.13)	0.04 (-0.05, 0.12)	0.03 (-0.07, 0.13)
Timing			
Unexposed	0 (Reference)	0 (Reference)	0 (Reference)
Early	-0.08 (-0.18, 0.02)	0.00 (-0.09, 0.10)	0.00 (-0.10, 0.10)
Mid	-0.12 (-0.28, 0.04)	-0.10 (-0.24, 0.04)	0.00 (-0.16, 0.16)
Late	0.03 (-0.13, 0.19)	-0.03 (-0.17, 0.11)	-0.04 (-0.2, 0.12)
**Small for gestational age (RR)**			
Ever/Never			
Never	1 (Reference)	1 (Reference)	1 (Reference)

© 2020 Huitfeldt A et al. *JAMA Network Open.*

Ever	1.12 (0.77, 1.62)	1.03 (0.70, 1.53)	0.86 (0.55, 1.35)
Timing			
Unexposed	1 (Reference)	1 (Reference)	1 (Reference)
Early	1.17 (0.79, 1.74)	0.95 (0.64, 1.41)	0.89 (0.59, 1.35)
Mid	1.40 (0.84, 2.34)	1.36 (0.82, 2.27)	1.01 (0.57, 1.78)
Late	1.14 (0.66, 1.97)	1.28 (0.77, 2.14)	1.26 (0.69, 2.31)
**Head circumference (cms)**			
Ever/Never			
Never	0 (Reference)	0 (Reference)	0 (Reference)
Ever	-0.2 (-0.3, 0)	-0.1 (-0.3, 0.1)	-0.2 (-0.4, 0.1)
Timing			
Unexposed	0 (Reference)	0 (Reference)	0 (Reference)
Early	-0.1 (-0.2, 0.1)	0.1 (-0.1, 0.2)	0.1 (-0.1, 0.3)
Mid	0.0 (-0.2, 0.3)	0.0 (-0.3, 0.4)	0.2 (-0.2, 0.7)
Late	-0.4 (-0.6, -0.1)	-0.3 (-0.6, -0.1)	-0.4 (-0.7, 0.0)
**Apgar Score Less than 7 at 5 minutes (RR)**			
Ever/Never			
Never	1 (Reference)	1 (Reference)	1 (Reference)
Ever	1.22 (0.52, 2.89)	1.23 (0.51, 2.98)	1.43 (0.58, 3.53)
Timing			
Unexposed	1 (Reference)	1 (Reference)	1 (Reference)
Early	0.83 (0.26, 2.7)	0.79 (0.22, 2.81)	0.70 (0.19, 2.59)
Mid	0.44 (0.05, 3.59)	0.46 (0.04, 4.96)	0.68 (0.05, 8.83)
Late	1.38 (0.32, 5.99)	1.39 (0.27, 7.22)	1.31 (0.20, 8.60)
**Respiratory distress (RR)**			
Ever/Never			
Never	1 (Reference)	1 (Reference)	1 (Reference)
Ever	1.51 (0.57, 4.01)	1.51 (0.57, 4.01)	1.45 (0.54, 3.86)
Timing			
Unexposed	1 (Reference)	1 (Reference)	1 (Reference)
Early	0.63 (0.14, 2.80)	0.64 (0.22, 1.89)	0.58 (0.20, 1.71)
Mid	1.43 (0.29, 7.15)	1.43 (0.40, 5.12)	1.16 (0.39, 3.48)
Late	1.52 (0.31, 7.45)	1.48 (0.30, 7.23)	1.30 (0.36, 4.64)

[1]Baseline covariates: BMI before conception, smoking, illicit drug use, alcohol intake, planned pregnancy, income, ongoing or completed education, adverse life events, sleeping and mental health problems, anxiety and LTH of MD.

[2]Time-varying covariates: Maternal symptoms of depression and anxiety during pregnancy, comedication use during pregnancy (NSAIDS, opioids, Paracetamol, antidepressants, antipsychotics and antiepileptics) and fever during pregnancy.

© 2020 Huitfeldt A et al. *JAMA Network Open.*

5b: Effect estimates with exposure definition restricted to z-hypnotics (N05CF) (n=82038, exposed: 282). 95% confidence intervals

	Crude analysis	Adjusted for baseline confounders[1]	Adjusted for baseline and time varying confounders[2]
**Gestational age (days)**			
Ever/Never			
Never	0 (Reference)	0 (Reference)	0 (Reference)
Ever	-1.4 (-2.7, 0.0)	-1.3 (-2.7, 0.1)	-0.7 (-2.2, 0.8)
Timing			
Unexposed	0 (Reference)	0 (Reference)	0 (Reference)
Early	-0.4 (-2.2, 1.3)	0.0 (-1.6, 1.7)	-0.5 (-2.1, 1.1)
Mid	0.8 (-1.9, 3.5)	-0.3 (-3.5, 3)	1.9 (-1.2, 5.1)
Late	-2.3 (-5, 0.5)	-1.7 (-4.9, 1.4)	-1.6 (-5.2, 2.1)
**Preterm delivery (RR)**			
Ever/Never			
Never	1 (Reference)	1 (Reference)	1 (Reference)
Ever	0.99 (0.57, 1.72)	0.96 (0.56, 1.62)	0.78 (0.43, 1.40)
Timing			
Unexposed	1 (Reference)	1 (Reference)	1 (Reference)
Early	1.02 (0.50, 2.10)	0.85 (0.40, 1.83)	0.86 (0.41, 1.78)
Mid	0.41 (0.09, 1.82)	0.67 (0.18, 2.48)	0.38 (0.08, 1.69)
Late	1.39 (0.49, 3.95)	1.18 (0.38, 3.64)	1.07 (0.28, 4.14)
**Birth weight (grams)**			
Ever/Never			
Never	0 (Reference)	0 (Reference)	0 (Reference)
Ever	-160.2 (-223.4, -97.0)	-108.2 (-169.8, -46.6)	-100.4 (-161.9, -38.8)
Timing			
Unexposed	0 (Reference)	0 (Reference)	0 (Reference)
Early	-121.8 (-203.3, -40.3)	-60.9 (-135.4, 13.5)	-43.4 (-123, 36.2)
Mid	-94 (-216.7, 28.8)	-85.2 (-221.2, 50.9)	-71.8 (-207.5, 63.8)
Late	-36.5 (-162.7, 89.7)	-30.6 (-159.6, 98.3)	-21.1 (-171.5, 129.3)
**Birth weight relative to gestational age and sex (z-scores)**			
Ever/Never			
Never	0 (Reference)	0 (Reference)	0 (Reference)
Ever	-0.25 (-0.37, -0.13)	-0.17 (-0.27, -0.07)	0.17 (0.06, 0.29)
Timing			
Unexposed	0 (Reference)	0 (Reference)	0 (Reference)
Early	-0.26 (-0.43, -0.10)	-0.15 (-0.28, -0.02)	-0.08 (-0.23, 0.06)
Mid	-0.19 (-0.44, 0.06)	-0.16 (-0.36, 0.04)	-0.24 (-0.48, -0.01)
Late	0.05 (-0.20, 0.31)	0.04 (-0.19, 0.26)	0.03 (-0.23, 0.29)
**Head circumference (cms)**			
Ever/Never			
Never	0 (Reference)	0 (Reference)	0 (Reference)
Ever	-0.3 (-0.5, -0.1)	-0.1 (-0.3, 0.1)	-0.1 (-0.3, 0.2)
Timing			
Unexposed	0 (Reference)	0 (Reference)	0 (Reference)

© 2020 Huitfeldt A et al. *JAMA Network Open.*

Early	-0.4 (-0.6, -0.1)	-0.2 (-0.4, 0.0)	-0.1 (-0.4, 0.1)
Mid	0.3 (-0.1, 0.7)	0.4 (-0.2, 0.9)	0.7 (-0.1, 1.5)
Late	-0.4 (-0.8, 0.0)	-0.3 (-0.7, 0.1)	-0.6 (-1.2, -0.1)
**Small for gestational age (RR)**			
Ever/Never			
Never	1 (Reference)	1 (Reference)	1 (Reference)
Ever	1.56 (1.06, 2.32)	1.32 (0.89, 1.95)	1.31 (0.82, 2.10)
Timing			
Unexposed	1 (Reference)	1 (Reference)	1 (Reference)
Early	1.65 (1.00, 2.72)	1.33 (0.81, 2.19)	1.04 (0.53, 2.06)
Mid	0.90 (0.38, 2.12)	0.90 (0.39, 2.10)	1.17 (0.47, 2.93)
Late	1.36 (0.62, 3.00)	1.33 (0.64, 2.80)	1.45 (0.67, 3.14)

[1]Baseline covariates: BMI before conception, smoking, illicit drug use, alcohol intake, planned pregnancy, income, ongoing or completed education, adverse life events, sleeping and mental health problems, anxiety and LTH of MD.

[2]Time-varying covariates: Maternal symptoms of depression and anxiety during pregnancy, comedication use during pregnancy (NSAIDS, opioids, Paracetamol, antidepressants, antipsychotics and antiepileptics) and fever during pregnancy.

The models for respiratory distress and Apgar score <7 at 5 minutes did not converge

© 2020 Huitfeldt A et al. *JAMA Network Open.*

5c: Effect estimates with exposure definition restricted to BZD-anxiolytics (N05BA) (n=82038, exposed: 373). 95% confidence intervals

	Crude analysis	Adjusted for baseline confounders[1]	Adjusted for baseline and time varying confounders[2]
**Gestational age (days)**			
Ever/Never			
Never	0 (Reference)	0 (Reference)	0 (Reference)
Ever	-2.5 (-3.7, -1.3)	-2.2 (-3.6, -0.7)	-2.4 (-4.3, -0.5)
Timing			
Unexposed	0 (Reference)	0 (Reference)	0 (Reference)
Early	0.1 (-1.1, 1.2)	0.4 (-0.8, 1.6)	0.2 (-1.1, 1.6)
Mid	-2.0 (-3.6, -0.3)	-2.3 (-4.5, -0.1)	-2.2 (-5, 0.6)
Late	-3.7 (-5.4, -2.0)	-3.4 (-5.7, -1.1)	-3.1 (-5.9, -0.3)
**Preterm delivery (RR)**			
Ever/Never			
Never	1 (Reference)	1 (Reference)	1 (Reference)
Ever	1.56 (1.07, 2.29)	1.44 (0.97, 2.13)	1.70 (1.10, 2.62)
Timing			
Unexposed	1 (Reference)	1 (Reference)	1 (Reference)
Early	0.76 (0.39, 1.46)	0.68 (0.35, 1.31)	0.71 (0.35, 1.41)
Mid	1.45 (0.73, 2.86)	1.42 (0.72, 2.8)	1.50 (0.63, 3.52)
Late	2.39 (1.34, 4.28)	2.37 (1.26, 4.44)	2.47 (1.16, 5.26)
**Birth weight (grams)**			
Ever/Never			
Never	0 (Reference)	0 (Reference)	0 (Reference)
Ever	-51.5 (-106.3, 3.3)	-33.1 (-92.7, 26.4)	-32.4 (-96.9, 32.1)
Timing			
Unexposed	0 (Reference)	0 (Reference)	0 (Reference)
Early	38.8 (-35.6, 113.3)	84.4 (11.2, 157.6)	49.5 (-32.6, 131.7)
Mid	-105.8 (-208.7, -2.9)	-97.9 (-215.9, 20.1)	-37.6 (-200.2, 124.9)
Late	-106.8 (-209.0, -4.7)	-135.1 (-257.9, -12.3)	-135 (-291.2, 21.1)
**Birth weight relative to gestational age and sex (z-scores)**			
Ever/Never			
Never	0 (Reference)	0 (Reference)	0 (Reference)
Ever	0.04 (-0.07, 0.15)	0.05 (-0.04, 0.14)	0.06 (-0.05, 0.17)
Timing			
Unexposed	0 (Reference)	0 (Reference)	0 (Reference)
Early	0.07 (-0.08, 0.22)	0.14 (0.01, 0.27)	0.09 (-0.05, 0.23)
Mid	-0.10 (-0.30, 0.11)	-0.07 (-0.25, 0.11)	0.06 (-0.15, 0.27)
Late	0.05 (-0.15, 0.26)	-0.05 (-0.22, 0.12)	-0.04 (-0.24, 0.16)
**Small for gestational age (RR)**			
Ever/Never			
Never	1 (Reference)	1 (Reference)	1 (Reference)
Ever	1.13 (0.75, 1.70)	1.04 (0.69, 1.58)	0.79 (0.50, 1.27)
Timing			
Unexposed	1 (Reference)	1 (Reference)	1 (Reference)

© 2020 Huitfeldt A et al. *JAMA Network Open.*

Early	0.90 (0.50, 1.62)	0.74 (0.42, 1.31)	0.72 (0.39, 1.32)
Mid	1.99 (1.08, 3.68)	1.82 (0.99, 3.35)	1.06 (0.46, 2.46)
Late	0.80 (0.36, 1.77)	1.00 (0.47, 2.14)	0.75 (0.24, 2.40)
**Head circumference (cms)**			
Ever/Never			
Never	0 (Reference)	0 (Reference)	0 (Reference)
Ever	-0.1 (-0.3, 0.0)	-0.0 (-0.2, 0.2)	-0.1 (-0.3, 0.1)
Timing			
Unexposed	0 (Reference)	0 (Reference)	0 (Reference)
Early	0.1 (-0.1, 0.3)	0.2 (0.0, 0.5)	0.2 (-0.1, 0.5)
Mid	-0.2 (-0.5, 0.1)	-0.2 (-0.6, 0.3)	0.0 (-0.6, 0.5)
Late	-0.3 (-0.6, 0.0)	-0.3 (-0.7, 0.1)	-0.4 (-0.9, 0.1)
**Apgar score less than 7 at 5 minutes (RR)**			
Ever/Never			
Never	1 (Reference)	1 (Reference)	1 (Reference)
Ever	1.13 (0.43, 3.00)	1.13 (0.42, 3.02)	1.08 (0.39, 3.00)
Timing			
Unexposed	1 (Reference)	1 (Reference)	1 (Reference)
Early	0.44 (0.06, 3.31)	0.40 (0.05, 3.5)	0.38 (0.05, 3.10)
Mid	0.75 (0.09, 6.29)	0.77 (0.07, 9.07)	0.91 (0.04, 19.06)
Late	2.15 (0.49, 9.41)	2.28 (0.43, 12.02)	1.61 (0.16, 16.61)
**Respiratory distress (RR)**			
Ever/Never			
Never	1 (Reference)	1 (Reference)	1 (Reference)
Ever	1.30 (0.42, 4.03)	1.31 (0.42, 4.06)	1.16 (0.37, 3.67)
Timing			
Unexposed	1 (Reference)	1 (Reference)	1 (Reference)
Early	1.05 (0.23, 4.87)	1.04 (0.35, 3.08)	1.30 (0.42, 3.98)
Mid	1.84 (0.33, 10.16)	1.82 (0.45, 7.35)	1.54 (0.38, 6.25)
Late	1.92 (0.37, 10.04)	1.86 (0.32, 10.66)	1.68 (0.33, 8.49)

[1]Baseline covariates: BMI before conception, smoking, illicit drug use, alcohol intake, planned pregnancy, income, ongoing or completed education, adverse life events, sleeping and mental health problems, anxiety and LTH of MD.

[2]Time-varying covariates: Maternal symptoms of depression and anxiety during pregnancy, comedication use during pregnancy (NSAIDS, opioids, Paracetamol, antidepressants, antipsychotics and antiepileptics) and fever during pregnancy.

© 2020 Huitfeldt A et al. *JAMA Network Open.*

eTable 6. Effects Estimates For Exposure To Benzodiazepines Or Benzodiazepine-like Hypnotic Drugs Stratified by Sex of Offspring

6a: Analysis stratified by sex of offspring. Effects estimates for exposure to BZDs or z-hypnotics. Boys (n=41987, exposed: 329). 95% confidence intervals

	**Crude analysis**	**Adjusted for baseline confounders[1]**	**Adjusted for baseline and time varying confounders[2]**
**Gestational age (days)**			
Ever/Never			
Never	0 (Reference)	0 (Reference)	0 (Reference)
Ever	-2.5 (-3.8, -1.3)	-2.6 (-4.1, -1.0)	-2.5 (-4.3, -0.8)
Timing			
Unexposed	0 (Reference)	0 (Reference)	0 (Reference)
Early	-0.8 (-2.6, 0.9)	-0.4 (-2.3, 1.4)	-0.5 (-2.6, 1.5)
Mid	-2.6 (-5.0, -0.2)	-3.3 (-6.7, 0.1)	-2.8 (-6.7, 1.1)
Late	-0.6 (-3.2, 1.9)	-0.2 (-3.4, 3.0)	-0.5 (-4.3, 3.3)
**Birth weight (grams)**			
Ever/Never			
Never	0 (Reference)	0 (Reference)	0 (Reference)
Ever	-97.5 (-169.8, -25.3)	-98.5 (-164.2, -32.7)	-97.5 (-169.8, -25.3)
Timing			
Unexposed	0 (Reference)	0 (Reference)	0 (Reference)
Early	-29.2 (-109.0, 50.6)	17.4 (-65.1, 99.9)	18.1 (-71.8, 108)
Mid	-129.3 (-238.3, -20.4)	-118.9 (-259.9, 22.2)	-112.6 (-269.7, 44.6)
Late	-87.0 (-205.0, 31.1)	-111.6 (-236.3, 13.1)	-103.0 (-256.4, 50.4)
**Birth weight relative to age and sex (z-scores)**			
Ever/Never			
Never	0 (Reference)	0 (Reference)	0 (Reference)
Ever	-0.12 (-0.24, -0.01)	0.05 (0.21, -0.11)	-0.08 (-0.18, 0.02)
Timing			
Unexposed	0 (Reference)	0 (Reference)	0 (Reference)
Early	-0.04 (-0.19, 0.12)	0.05 (-0.08, 0.18)	0.05 (-0.09, 0.19)
Mid	-0.08 (-0.30, 0.13)	-0.07 (-0.26, 0.12)	-0.08 (-0.29, 0.14)
Late	-0.19 (-0.42, 0.05)	-0.24 (-0.43, -0.06)	-0.20 (-0.43, 0.04)

[1]Baseline covariates: BMI before conception, smoking, illicit drug use, alcohol intake, planned pregnancy, income, ongoing or completed education, adverse life events, sleeping and mental health problems, anxiety and LTH of MD.

[2]Time-varying covariates: Maternal symptoms of depression and anxiety during pregnancy, comedication use during pregnancy (NSAIDS, opioids, Paracetamol, antidepressants, antipsychotics and antiepileptics) and fever during pregnancy.

© 2020 Huitfeldt A et al. *JAMA Network Open.*

6b: Analysis stratified by sex of offspring. Effects estimates for exposure to BZDs or z-hypnotics. Girls (n=40051, exposed: 350). 95% confidence intervals

	Crude analysis	Adjusted for baseline confounders[1]	Adjusted for baseline and time varying confounders[2]
**Gestational age (days)**			
Ever/Never			
Never	0 (Reference)	0 (Reference)	0 (Reference)
Ever	-2.4 (-3.6, -1.2)	-2.1 (-3.5, -0.7)	-2.0 (-3.6, -0.4)
Timing			
Unexposed	0 (Reference)	0 (Reference)	0 (Reference)
Early	0.8 (-0.8, 2.4)	1.1 (-0.4, 2.6)	1.5 (-0.1, 3.2)
Mid	-1.5 (-3.8, 0.9)	-1.2 (-4.0, 1.6)	-2 (-5.5, 1.5)
Late	-6.0 (-8.2, -3.8)	-5.9 (-9.1, -2.7)	-5.5 (-9.0, -2.0)
**Birth weight (grams)**			
Ever/Never			
Never	0 (Reference)	0 (Reference)	0 (Reference)
Ever	-82.1 (-136.9, -27.3)	-58.0 (-118, 2.1)	-59.8 (-121.6, 2.0)
Timing			
Unexposed	0 (Reference)	0 (Reference)	0 (Reference)
Early	-17.5 (-90.3, 55.3)	37.0 (-32.2, 106.1)	53.3 (-25.2, 131.8)
Mid	-100.4 (-207.7, 6.9)	-90.7 (-202.4, 21.1)	-93.5 (-242.4, 55.5)
Late	-88.7 (-188.9, 11.5)	-121.3 (-251.5, 8.9)	-150.4 (-287.5, -13.3)
**Birth weight relative to age and sex (z-scores)**			
Ever/Never			
Never	0 (Reference)	0 (Reference)	0 (Reference)
Ever	-0.04 (-0.16, 0.07)	0 (-0.1, 0.09)	-0.01 (-0.13, 0.1)
Timing			
Unexposed	0 (Reference)	0 (Reference)	0 (Reference)
Early	-0.11 (-0.26, 0.04)	0 (-0.13, 0.13)	0.01 (-0.13, 0.16)
Mid	-0.14 (-0.37, 0.08)	-0.13 (-0.32, 0.05)	-0.07 (-0.32, 0.17)
Late	0.17 (-0.04, 0.39)	0.1 (-0.08, 0.29)	0 (-0.22, 0.21)

[1]Baseline covariates: BMI before conception, smoking, illicit drug use, alcohol intake, planned pregnancy, income, ongoing or completed education, adverse life events, sleeping and mental health problems, anxiety and LTH of MD.

[2]Time-varying covariates: Maternal symptoms of depression and anxiety during pregnancy, comedication use during pregnancy (NSAIDS, opioids, Paracetamol, antidepressants, antipsychotics and antiepileptics) and fever during pregnancy.

© 2020 Huitfeldt A et al. *JAMA Network Open.*

eTable 7. Effects Estimates for Exposure to Benzodiazepines or Benzodiazepine-like Hypnotic Drugs in Dataset Restricted to First-Time Participants in the Norwegian Mother, Father and Child Cohort Study

	Crude analysis	Adjusted for baseline confounders[1]	Adjusted for baseline and time varying confounders[2]
**Gestational age (days)**			
Ever/Never			
Never	0 (Reference)	0 (Reference)	0 (Reference)
Ever	-2.5 (-3.5, -1.6)	-2.4 (-3.5, -1.2)	-2.3 (-3.6, -1.0)
Timing			
Unexposed	0 (Reference)	0 (Reference)	0 (Reference)
Early	-0.1 (-1.4, 1.2)	0.2 (-1.0, 1.5)	0.2 (-1.2, 1.6)
Mid	-1.9 (-3.7, 0.0)	-2.3 (-4.8, 0.3)	-1.8 (-5.0, 1.3)
Late	-3.8 (-5.7, -2.0)	-3.5 (-6.1, -0.8)	-3.5 (-6.4, -0.6)
Duration			
0	0 (Reference)	0 (Reference)	0 (Reference)
1	-2.5 (-3.7, -1.3)	-2.5 (-4.0, -1.0)	-2.7 (-4.3, -1.1)
2	-1.9 (-4.3, 0.6)	-1.4 (-4.5, 1.7)	-0.8 (-4.3, 2.7)
3+	-3.0 (-5.1, -1.0)	-2.7 (-4.8, -0.6)	-2.2 (-4.8, 0.4)
**Preterm delivery (RR)**			
Ever/Never			
Never	1 (Reference)	1 (Reference)	1 (Reference)
Ever	1.55 (1.16, 2.09)	1.46 (1.07, 1.98)	1.44 (1.04, 2.01)
Timing			
Unexposed	1 (Reference)	1 (Reference)	1 (Reference)
Early	0.80 (0.49, 1.31)	0.72 (0.43, 1.22)	0.66 (0.37, 1.19)
Mid	1.06 (0.58, 1.95)	1.15 (0.63, 2.13)	1.33 (0.68, 2.61)
Late	2.55 (1.60, 4.09)	2.38 (1.39, 4.06)	2.24 (1.24, 4.05)
Duration			
0	1 (Reference)	1 (Reference)	1 (Reference)
1	1.57 (1.08, 2.27)	1.52 (1.04, 2.20)	1.22 (0.56, 2.67)
2	1.76 (0.86, 3.59)	1.57 (0.76, 3.24)	1.48 (0.69, 3.17)
3+	1.37 (0.70, 2.68)	1.21 (0.61, 2.42)	1.51 (0.95, 2.42)
**Birth weight relative to gestational age and sex (z-scores)**			
Ever/Never			
Never	0 (Reference)	0 (Reference)	0 (Reference)
Ever	-0.08 (-0.17, 0.01)	-0.05 (-0.12, 0.03)	-0.05 (-0.13, 0.03)
Timing			
Unexposed	0 (Reference)	0 (Reference)	0 (Reference)
Early	-0.07 (-0.18, 0.05)	0.02 (-0.07, 0.12)	0.03 (-0.07, 0.13)
Mid	-0.05 (-0.22, 0.13)	-0.03 (-0.17, 0.11)	0.00 (-0.17, 0.16)
Late	-0.05 (-0.23, 0.12)	-0.13 (-0.26, 0.01)	-0.12 (-0.29, 0.04)
Duration			
0	0 (Reference)	0 (Reference)	0 (Reference)
1	-0.06 (-0.17, 0.06)	-0.02 (-0.11, 0.07)	-0.04 (-0.14, 0.06)
2	-0.12 (-0.35, 0.12)	-0.08 (-0.26, 0.10)	-0.09 (-0.28, 0.11)

© 2020 Huitfeldt A et al. *JAMA Network Open.*

3+	-0.14 (-0.33, 0.05)	-0.1 (-0.25, 0.06)	0.09 (-0.11, 0.28)
**Small for gestational age (RR)**			
Ever/Never			
Never	1 (Reference)	1 (Reference)	1 (Reference)
Ever	1.07 (0.78, 1.48)	0.96 (0.69, 1.32)	0.83 (0.58, 1.20)
Timing			
Unexposed	1 (Reference)	1 (Reference)	1 (Reference)
Early	1.19 (0.80, 1.78)	0.97 (0.66, 1.44)	0.92 (0.61, 1.39)
Mid	0.82 (0.43, 1.58)	0.80 (0.44, 1.47)	0.57 (0.28, 1.16)
Late	1.31 (0.74, 2.32)	1.45 (0.87, 2.42)	1.38 (0.75, 2.53)
Duration			
0	1 (Reference)	1 (Reference)	1 (Reference)
1	0.88 (0.56, 1.38)	0.80 (0.52, 1.25)	0.80 (0.50, 1.28)
2	1.02 (0.43, 2.38)	0.90 (0.38, 2.16)	0.95 (0.39, 2.36)
3+	1.66 (0.97, 2.85)	1.43 (0.84, 2.45)	0.83 (0.40, 1.73)
**Birthweight (grams)**			
Ever/Never			
Never	0 (Reference)	0 (Reference)	0 (Reference)
Ever	-109.5 (-153.7, -65.2)	-80.8 (-129, -32.6)	-79.8 (-131.4, -28.3)
Timing			
Unexposed	0 (Reference)	0 (Reference)	0 (Reference)
Early	-23.4 (-81.3, 34.6)	28.5 (-28.3, 85.3)	31.4 (-31.7, 94.5)
Mid	-75.8 (-161.6, 10.0)	-72.5 (-174.4, 29.5)	-63.2 (-188.3, 61.9)
Late	-137.7 (-223.2, -52.1)	-159.2 (-261.3, -57.0)	-148.1 (-263.3, -32.8)
Duration			
0	0 (Reference)	0 (Reference)	0 (Reference)
1	-98.5 (-154.2, -42.8)	-75.1 (-137.6, -12.5)	-90.9 (-157.5, -24.3)
2	-126.6 (-240.1, -13.1)	-80 (-202.6, 42.6)	-71.5 (-209.1, 66.1)
3+	-128.9 (-222.6, -35.1)	-97.9 (-187.7, -8.1)	-41.9 (-163.3, 79.6)
**Head circumference (cms)**			
Ever/Never			
Never	0 (Reference)	0 (Reference)	0 (Reference)
Ever	-0.2 (-0.4, -0.1)	-0.2 (-0.4, 0.0)	-0.1 (-0.3, 0.1)
Timing			
Unexposed	0 (Reference)	0 (Reference)	0 (Reference)
Early	-0.1 (-0.3, 0.1)	0.1 (-0.1, 0.3)	0.1 (-0.1, 0.3)
Mid	0.1 (-0.2, 0.3)	0.1 (-0.3, 0.5)	0.2 (-0.3, 0.7)
Late	-0.4 (-0.7, -0.2)	-0.5 (-0.8, -0.2)	-0.5 (-0.9, -0.1)
Duration			
0	0 (Reference)	0 (Reference)	0 (Reference)
1	-0.2 (-0.4, 0.0)	-0.1 (-0.3, 0.1)	-0.1 (-0.4, 0.1)
2	-0.2 (-0.5, 0.2)	0.0 (-0.5, 0.5)	0.0 (-0.5, 0.5)
3+	-0.3 (-0.6, 0.0)	-0.1 (-0.4, 0.2)	0.1 (-0.3, 0.5)
**Apgar Score Less than 7 at 5 minutes (RR)**			
Ever/Never			
Never	1 (Reference)	1 (Reference)	1 (Reference)
Ever	1.01 (0.46, 2.26)	0.98 (0.43, 2.2)	1.12 (0.48, 2.61)
Timing			
Unexposed	1 (Reference)	1 (Reference)	1 (Reference)

© 2020 Huitfeldt A et al. *JAMA Network Open.*

Early	0.49 (0.12, 2.06)	0.45 (0.09, 2.13)	0.35 (0.07, 1.67)
Mid	0.43 (0.05, 3.52)	0.43 (0.04, 4.61)	0.55 (0.05, 5.90)
Late	2.41 (0.73, 7.97)	2.46 (0.64, 9.43)	3.06 (0.8, 11.75)
**Respiratory distress (RR)**			
Ever/Never			
Never	1 (Reference)	1 (Reference)	1 (Reference)
Ever	0.80 (0.26, 2.49)	0.8 (0.26, 2.48)	0.69 (0.22, 2.19)
Timing			
Unexposed	1 (Reference)	1 (Reference)	1 (Reference)
Early	0.43 (0.06, 2.96)	0.67 (0.23, 1.97)	0.58 (0.19, 1.73)
Mid	1.77 (0.25, 12.35)	1.52 (0.42, 5.55)	1.16 (0.37, 3.62)
Late	1.21 (0.17, 8.42)	1.49 (0.30, 7.44)	1.51 (0.42, 5.39)
Duration			
0	1 (Reference)	1 (Reference)	1 (Reference)
1	0.43 (0.06, 2.96)	0.43 (0.06, 3.02)	0.28 (0.04, 2.00)
2	1.77 (0.25, 12.35)	1.90 (0.26, 13.65)	0.96 (0.13, 7.38)
3+	1.21 (0.17, 8.42)	1.16 (0.16, 8.44)	1.21 (0.16, 8.90)

[1]Baseline covariates: BMI before conception, smoking, illicit drug use, alcohol intake, planned pregnancy, income, ongoing or completed education, adverse life events, sleeping and mental health problems, anxiety and LTH of MD.

[2]Time-varying covariates: Maternal symptoms of depression and anxiety during pregnancy, comedication use during pregnancy (NSAIDS, opioids, Paracetamol, antidepressants, antipsychotics and antiepileptics) and fever during pregnancy.

© 2020 Huitfeldt A et al. *JAMA Network Open.*
